# Food-Grade Oleogels: Trends in Analysis, Characterization, and Applicability

**DOI:** 10.3390/gels9050386

**Published:** 2023-05-06

**Authors:** Simona Perța-Crișan, Claudiu-Ștefan Ursachi, Bianca-Denisa Chereji, Iolanda Tolan, Florentina-Daniela Munteanu

**Affiliations:** Faculty of Food Engineering, Tourism and Environmental Protection, “Aurel Vlaicu” University of Arad, 2-4 E. Drăgoi Str., 310330 Arad, Romania; simona.perta-crisan@uav.ro (S.P.-C.); claudiu.ursachi@uav.ro (C.-Ș.U.); bianca.chereji@yahoo.com (B.-D.C.); iolanda.tolan@uav.ro (I.T.)

**Keywords:** food-grade oleogels, oleogelators, microscopy, rheology, texture analysis, oxidative stability, food applicability

## Abstract

Currently, a large number of scientific articles can be found in the research literature in the field focusing on the use of oleogels for food formulation to improve their nutritional properties. The present review focuses on the most representative food-grade oleogels, highlighting current trends in terms of the most suitable methods of analysis and characterization, as well as trends in their application as substitutes for saturated and trans fats in foods. For this purpose, the physicochemical properties, structure, and composition of some oleogelators are primarily discussed, along with the adequacy of oleogel incorporation for use in edible products. Analysis and characterization of oleogels by different methods are important in the formulation of innovative foods, and therefore, this review discusses the most recent published results regarding their microstructure, rheological and textural properties, and oxidative stability. Last but not least, issues related to the sensory properties of oleogel-based foods are discussed, highlighting also the consumer acceptability of some of them.

## 1. Introduction

Oleogelation is a relatively new and innovative technology that has attracted attention in the food industry due to its ability to convert liquid oils into semi-solid gels without the need for chemical modifications or additives [1].

Oleogels are unique semi-solid materials consisting of a network of structuring molecules that entrap liquid oil, resulting in a gel-like texture. They have gained increasing interest in the food industry due to their ability to mimic the functionality of fats and deliver bioactive compounds, offering potential solutions to various challenges and opportunities for the creation of innovative food products [2]. Recently, research in the field of oleogels has focused on investigating new molecules or combinations of molecules that can act as structuring agents, as well as developing new methods for gelation. This includes exploring different types of structuring agents, such as waxes [3,4,5,6,7,8,9,10] and polymers [11,12], and investigating their properties and interactions with liquid oils to create stable and functional oleogels [13,14]. Scientists are also investigating the use of new techniques, such as micro- and nano-structuring [15,16,17,18,19,20,21,22,23,24], to modulate oleogel properties and create novel structures with specific functionality [17,25,26,27,28].

From a technological perspective, oleogels are being explored as a way to overcome challenges in food product development. For example, they can be used in food products as a replacement or for the reduction of solid fats, such as trans fats or hydrogenated fats, to create healthier options without compromising their texture, mouthfeel, and stability [1,29,30,31,32,33,34,35,36]. Oleogels can also be used to encapsulate and deliver bioactive compounds, such as antioxidants or flavors, in food products, providing enhanced stability and controlled-release properties [2,37,38,39,40,41,42,43,44]. Additionally, oleogels can offer new possibilities for product innovation by creating unique textures, structures, and sensory experiences in food products [45].

One of the advantages of food-grade oleogels is their ability to reduce or eliminate trans fats, which are known to be harmful to health, while maintaining desirable functional properties of fats, such as mouthfeel, creaminess, and stability. Oleogels can also be formulated to have specific melting properties, which can be beneficial in various food applications, such as controlling the release of flavors or preventing oil migration.

The relationship between dietary fats and health, particularly cardiovascular health, has been a topic of ongoing research and debate. While there is evidence from clinical trials suggesting that replacing saturated fats with polyunsaturated fats and essential fatty acids may reduce the risk of coronary heart disease, recent studies have questioned the simple explanation of the relationship between dietary saturated fats and cardiovascular disease [46,47]. As a result, current recommendations emphasize the importance of critically evaluating the scientific understanding of dietary fats and health [48]. 

Given these concerns about saturated fats and trans fats in the diet, there has been increasing interest among manufacturers and scientists in finding alternative ways to modify or structure liquid oils to mimic the functionality of fats in food products without changing the nutritional profile of the oil. This has led to the exploration of oleogels as a potential solution, as they offer a unique approach to structuring liquid oils without the use of saturated or trans fats [36,49,50].

Recent food applications of oleogels have shown promise in the area of bioactive delivery, where oleogels can be used as carriers for bioactive compounds such as vitamins, antioxidants, and nutraceuticals [2,51,52,53]. Oleogels can protect bioactive compounds from degradation, control their release, and enhance their bioavailability, making them suitable for functional food applications. Oleogels as a promising class of structured edible oils with unique properties and versatile applications in the food industry have also found applications in confectionery, bakery, spreads, margarines, and meat products, due to their ability to improve texture, reduce saturated fat content, and provide clean-label options. 

Oleogels can be formulated using structuring agents that are Generally Recognized as Safe (GRAS) and can help replace solid fats in food products without compromising their sensory properties or functionality. This aligns with the current recommendations to limit saturated fat intake and remove trans fats from the diet while still providing functional and palatable food products to consumers.

The present review discusses the most used oleogelators for the preparation of food-grade oleogels, along with specific analyses that can be used for the characterization of food oleogels and their behavior as food components. These analyses can provide insights into the physical, chemical, rheological, and structural properties of oleogels, which are important for understanding their performance in food applications [3,11,54,55,56,57,58,59,60,61,62,63,64,65,66,67]. 

Overall, the food industry is increasingly exploring the use of oleogels in various food products to improve their texture, stability, sensory attributes, and nutritional profile. The unique functional properties of oleogels make them attractive for a wide range of applications in the food industry, offering opportunities for innovation and the development of healthier and more desirable food products.

## 2. Oleogelators

**Figure 1:**
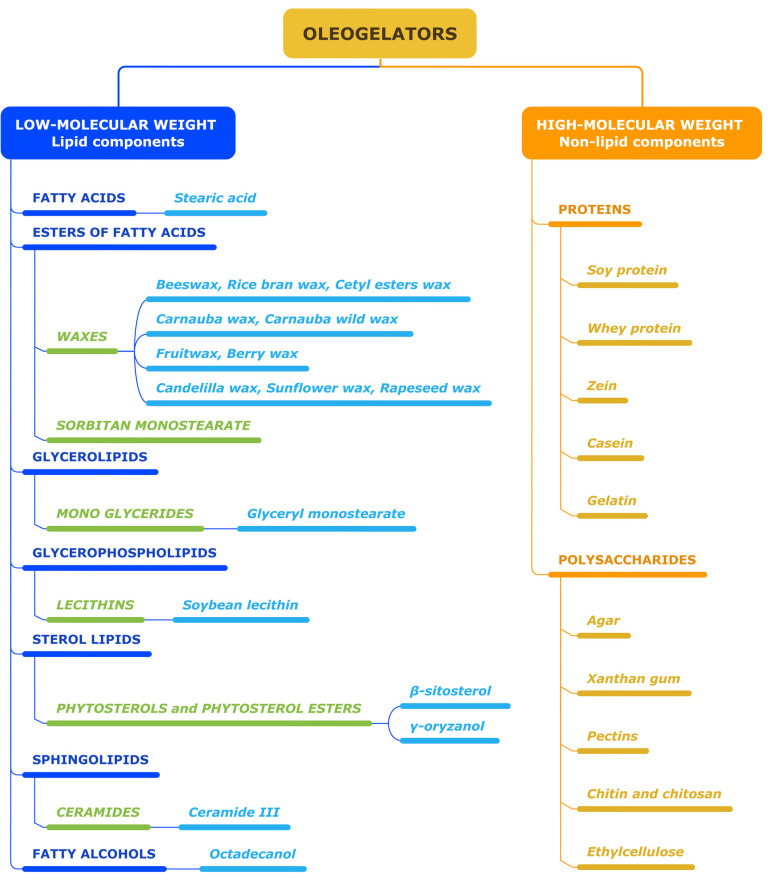
The most used oleogelators for food-grade oleogels.

Oleogelators are substances that are used to create gels or semi-solid systems in oil-based formulations. They have an important role in changing the viscosity or thickness of the oil phase, resulting in a gel-like consistency. Various types of oleogelators can be used in different applications, as shown in Figure 1.

The properties and performance of oleogelators can vary depending on their chemical structure, concentration, and formulation conditions, and they are often chosen based on the specific requirements of the intended application. 

The growing number of compounds used for structuring vegetable oils calls for their categorization. Thus, two large categories are identified: low-molecular-weight oil and polymeric or high-molecular-weight oil gelators (Table 1).

### 2.1. Low-Molecular-Weight Oleogelators

Low-molecular-weight oil gelators include components present in waxes from natural sources (i.e., long-chain esters, fatty acids, fatty alcohols, hydrocarbons), which build three-dimensional crystal networks, and components present in phytosterols (i.e., sterols and sterol esters) that develop self-assembled fibrillary networks [82].

#### 2.1.1. Waxes

Waxes are biosynthesized crystalline solids obtained from plants (e.g., carnauba wax, candelilla wax) or animal sources (e.g., beeswax) [72] with one particular lipidic composition. In general, waxes are mixtures of medium- or long-chain fatty acids and long-chain fatty alcohols in free or esterified form (wax esters). They can also contain hydrocarbons, ketones, and sterols. Only beeswax and candelilla wax have a higher hydrocarbon content. Sunflower wax and rice bran wax can be considered single-component waxes because almost 90% of their composition represents wax esters [68]. Being from natural sources, the chemical composition of waxes can vary across their origins [68,70]. When the crystalline network of waxes traps the liquid oils, oleogels are formed [83]. The wax esters lead to strong but brittle oleogels, and the free fatty acids and hydrocarbons lead to consistent and stable gels. Free fatty alcohols have also an important role in oil structuring [68].

##### Beeswax

Beeswax is one crystalline solid with a characteristic honey odor, produced by the *Apis mellifera*, *Apis cerana, Apis florae*, and other bee species for honeycomb construction [69,84,85]. Two types of waxes are obtained from *Apis mellifera* bees: yellow beeswax and white beeswax. The first type is a yellow or yellow-brown solid that becomes pliable at about 35 °C. The second type is a white or yellowish-white solid, which in a thin layer is translucent and is obtained by bleaching yellow beeswax. Beeswax is insoluble in water, weak-soluble in alcohol, and very soluble in chloroform and ether. Its melting range is between 62 and 65 °C, and its specific gravity is about 0.96 [69,75,84,86]. In the USA, beeswax has GRAS (Generally Recognized as Safe) grade, and in the EU, it is an approved food additive (E number E901) [75,87,88].

The composition of beeswaxes may vary depending on the honeybee species, the geographical origin, and the age of the wax [75,84]. Thus, the reported values have fairly wide ranges. Chemically, beeswax is a complex mixture of fatty acid esters (mono-, hydroxy mono-, and di-esters) (58–71%), hydrocarbons (alkanes and alkenes) (12–26%), free fatty acids (9–15%), free fatty alcohols (1–6%), and other minor compounds [68,69,70,71,72]. Hydrocarbons have long chains with odd carbon numbers, within the range of 27–33. Esters consist of long-chain alcohols and diols with even carbon numbers (24–32), and long-chain fatty acids and hydroxy acids have even carbon numbers (16–24) [71].

Due to its composition and low cost, beeswax is one of the most studied oil-structuring agents [33,68,70,71,72,89,90,91].

##### Carnauba Wax

Carnauba wax (named also palm wax or Brazilian wax) is produced by the leaf buds and leaves of the carnauba wax palm, *Copernicia cerifera Martius,* from Brazil. The marketed product is a purified wax. It occurs as light brown to yellowish flakes or hard and brittle solids. It has a weakly aromatic odor at room temperature and a characteristic hay smell in the molten state [85]. Carnauba wax is one of the hardest and highest-melting-point natural waxes with a melting range between 82 and 86 °C and specific gravity of about 0.997. It is insoluble in water, weak-soluble in alcohol, and soluble in chloroform and ether [6,85]. The EU approved it as a food additive (E number E903), and the USA gave GRAS grade to it [75,87].

Chemically, carnauba wax is a complex mixture of fatty esters (62–85%), free fatty alcohols (10–30%), free fatty acids (3–6%), hydrocarbons (paraffins), and triterpene diols. The esters of carnauba wax are mainly aliphatic esters and diesters of cinnamic acid. The free acids are aliphatic, aromatic, and ω-hydroxycarboxylic types [6,68,72]. Due to its high content of fatty esters and its inert and stable components, carnauba wax is widely used for oil structuring [9,54,68,70,72,90,91,92].

##### Sunflower Wax

Sunflower wax is a crystalline solid extracted from the seeds and seed hull of sunflower *(Helianthus annuus)* [70,93]. The marketed sunflower wax is a hard and light yellow solid [84], with a melting range between 70 and 80 °C [93,94]. Above 40 °C, sunflower wax is lipophilic. When cooled to below 40 °C, it acquires weak hydrophilic characteristics and crystallizes [93]. More than 95% of the composition is esters, and it can be considered a single-component wax [70]. It also contains approx. 3% free fatty acids, 0.3% free fatty alcohol, and 0.17% hydrocarbons [68]. The esters of sunflower wax consist predominantly of chains with 44–50 carbons, and their major free fatty acids have chains with 16–24 carbons. Sunflower wax is declared the best wax-type oil gelling agent, and sunflower wax oleogels are accepted as the gold standard for plant wax-type oleogels [83]. Thus, *Yilmaz E.* et al. reported that the C* (%) of sunflower wax in sunflower oil is 1% [83]. The oleogels formed with sunflower wax have been much studied [83,90,93,95,96].

##### Rice Bran Wax

Rice bran wax is a yellow, hard, crystalline solid obtained as a by-product from rice bran oil derived from the bran, which is a by-product of rice *(Oryza sativa)* milling [84,97,98]. Rice bran contains 10–12% lipidic compounds [99] and is a rich source of phytosterols and tocopherols [100]. Rice bran wax has a high melting range between 77 and 81 °C [97]. Almost 93% of its composition is high-molecular-weight C48-C64 monoesters. Rice bran wax is also named a one-component wax, similar to sunflower wax [68]. The other components are free fatty acids, less than 1% free fatty alcohols, and hydrocarbons. It is also a rich source of oryzanols [100]. Rice bran wax can form oleogel at the addition of less than 1% to the oil. In oils, rice bran wax forms long, thin needle-like crystals [97]. 

#### 2.1.2. Phytosterols and Phytosterol Esters

Phytosterols or plant sterols are a large group of steroid-type alcohols that exist in free or esterified forms, named ester phytosterols or conjugate phytosterols. Phytosterols and their esters are biosynthesized exclusively by plants [101]. The core of this compound is one tetracyclic skeleton consisting of three cyclohexanes and one cyclopentane ring, with one aliphatic side chain. Commercially, phytosterols and their esters are extracted from the unsaponifiable fraction of lipid-rich plant oils such as soy, canola (rapeseed), corn, sunflower, cotton seed, flax seed, pumpkin seed, peanut, olive, etc. [101,102,103], or cereal by-products such as corn fiber, wheat germ, wheat bran, rice bran, oat bran, or oat hull. Tall oil, a by-product of wood pulps, is a phytosterol source too [104]. The major compounds of phytosterols are β-sitosterol (3β)-Stigmast-5-en-3-ol, stigmasterol (3β)-Stigmasta-5,22-dien-3-ol, brassica sterol (3β)-Ergosta-5,22-dien-3-ol, and campesterol (3β)-Ergost-5-en-3-ol [102]. Oryzanol is a sterol ester that occurs naturally mainly in rice bran oil [105]. Apart from the biosynthesized ones, phytosterol esters can also be obtained through the interesterification of phytosterols such as sitosterol, with fatty acids from vegetable oils such as canola or rapeseed [105]. Phytosterol extracts and phytosterol ester extracts are available as nutritional supplements marketed as dietary supplements [106]. In the USA, in the Chemical and Technical Assessment reported in 2012 by JECFA [106], phytosterols, phytostanols, and their esters are Generally Recognized as Safe (GRAS). In the EU, stigmasterol-rich plant sterols are included in the food additive list, with E number E499. Stigmasterol-rich plant sterols must contain over 85% stigmasterol and other sterols. Mixtures of phytosterols (e.g., β-sitosterol, stigmasterol) and their esters (γ-oryzanol) have the oil-structuring ability. They form hazy transparent oleogels [105,107].

##### β-Sitosterol

β-sitosterol C_29_H_50_O, with a molecular weight of 414.72 g/mol, is one abundant representative of phytosterols [108], and its molecule is bioactive [101]. β-sitosterol is a white to off-white waxy powder, with a characteristic odor and melting range between 136 and 142 °C [101,109]. Above 110 °C, β-sitosterol is completely dissolved in water. When cooling down, up to 80 °C, it crystallizes in anhydrous form, between 45 and 80 °C, it crystallizes as a hemihydrate, and below 45 °C, it is a monohydrate. β-sitosterol crystals are organized in bilayers of approximately 3.8 nm thickness. Side-products of vegetable oils extracted from soybeans, peanuts, avocadoes, rice bran, wheat germ, corn, rapeseed [101,102,109], or tall oil [110,111] are rich sources of β-sitosterol. Finally, microalgal species (diatome, chlorophyte, and haptophytes) were studied too as alternative sources of β-sitosterol [52]. β-sitosterol is one of the components of the approved food additive (E number E499) [112]. Recently, in addition to its beneficial effects on health, β-sitosterol has also been studied as a gelling agent for vegetable oils. Mixtures of β-sitosterol with γ-oryzanol were used for the gelation of different vegetable oils. Arjen Bot et al. reported that these two compounds, used alone, do not have oil-structuring ability [105].

##### γ-Oryzanol

γ-oryzanol is a blend of sterol esters that naturally occur in cereals and plant oils. It is obtained from the unsaponifiable fraction of rice bran oil (*Oryza sativa*) [113]. Values within 0.9–2.9% have been reported [59,114]. The concentrations of γ-oryzanol depend significantly on the type of oils and genetic and environmental factors. It has also been shown in barley [100]. γ-Oryzanol is a white or slightly yellowish tasteless crystalline powder with little or no odor and has a melting point of 135.7–138.5 °C [114]. It is hardly soluble in water, slightly soluble in diethyl ether and n-heptane, practically soluble in chloroform [99], and highly soluble in vegetable oils [98]. γ-oryzanol is obtained from *Oryza sativa Japonica* (rice bran and germ) as a refined, dried, white, or light yellowish extract. γ-Oryzanol is a mixture of ferulic acid esters of phytosterols and triterpenoids. About 80% of γ-oryzanol is represented by cycloartenyl ferulate, 24-methylenecycloartanyl ferulate, and campesteryl ferulate [59,99,100]. In a mixture with sterols (especially β-sitosterol), γ-oryzanol is used as an oil-structuring agent [98]. Dissolved in plant oils, the molecules of the mixture of γ-oryzanol and β-sitosterol, in a 60%:40% *w*/*w* (1:1 mol/mol) ratio, can stack and form self-assembled fibrils by hydrogen bonds. These fibrils form three-dimensional ribbon-like tubules with a 7.2 nm diameter. The wall thickness of ribbons is 0.8 nm. This thickness corresponds to the size of the sterane core of β-sitosterol and γ-oryzanol [98,115]. A transparent, firm gel occurs when the oil is trapped in the network formed by the aggregation of these tubes. The formed gels are clear and slightly cloudy [105]. The literature on the conditions of formation and stability of this type of oleogels is rich [21,59,105,115,116]. 

#### 2.1.3. Fatty Acids and Monoglycerides

Free fatty acids (stearic acid, palmitic acid, oleic acid) and monoglycerides (monostearin, monopalmitin, glycerol monolaurate, glycerol monobehenate) represent two groups of oleogelators that can be used to create stable gels in lipid-based systems [64,117,118]. Due to the different chemical groups and structures, they form different molecular arrangements and architectures, leading to various textural or mechanical properties of oleogels.

The advantages of monoglycerides as oleogelators are that they can crystallize and form strong networks even at low concentrations (3%) [1]. As shown in numerous studies, the crystallization mechanism of monoacylglycerols consists of the formation and growth of a laminar crystal network, and some of the oleogel properties (thermal stability, mechanical properties) can be controlled by the chain length, molecular weight, and concentration of monoacylglycerols [2,3,4,5,6].

### 2.2. High-Molecular-Weight Oleogelators

High-molecular-weight oleogelators include proteins and polysaccharides. They can be used separately or in different combinations to form a three-dimensional network stabilized by hydrogen bonds, electrostatic interactions, and hydrophobic interactions between molecules, where liquid oils are entrapped [119]. These two classes of polymers have drawn increasing attention since they are food-grade, inexpensive, and readily available as by-products of different food industry branches [8].

#### 2.2.1. Proteins

Based on their shape, protein oleogelators may be divided into two classes: globular and fibrous. Globular proteins can form a gel-like structure due to their ability to create cross-links or aggregates [120,121]. The gelation process typically involves heating a solution of globular proteins, which causes them to denature and unfold. As the proteins unfold, amino acids begin to interact with each other, forming aggregates and cross-links that create a three-dimensional network. This network traps water and other molecules, forming a gel-like substance [120,122,123]. On the other hand, fibrous proteins, such as gelatin, can form a gel when they are heated and then cooled. The gelation mechanism involves the reversible unfolding and refolding of protein chains, as well as the formation of physical cross-links between protein molecules [124]. In the last years, several studies on protein-based oleogels have been performed.

##### Soy Protein

Soy protein is considered a good option for use in the production of vegetable protein, due to its excellent gelation properties. It has been widely used since 1990 as an excellent animal protein substitute. Furthermore, soy protein is a high-quality protein that contains all the essential amino acids and can reduce cholesterol levels and the risk of cardiovascular diseases [125].

A protein oleogel system was fabricated using linseed oil and soy protein isolate with different concentrations of highland barley *β*-glucan by using the emulsion-templated approach [126]. It was found that linseed oil soy-based oleogels formed a strong 3D network with a reduced lipid oxidation rate. Furthermore, with the addition of highland barley *β*-glucan, the oil droplets became smaller and more uniformly distributed in the network.

##### Whey Protein

Whey protein is a type of protein that is derived from milk as a by-product of the cheese-making process. It is a complete protein, which means that it contains all nine essential amino acids [127]. Whey protein isolate is a purified form of whey protein that contains a higher percentage of protein and small amounts of other substances such as fat and carbohydrates [128]. Meissner et al. [129] obtained a stable oleogel system using safflower oil gelated with whey protein by the solvent-exchange method. The results also showed that the presence of protein promoted the decomposition of lipid oxidation products in the prepared oleogels. 

##### Zein

Zein is a plant protein that is commonly found in corn. It is a type of prolamin protein, which is a storage protein found in the seeds of cereal crops. The amino acid profile of zein is made of glutamic acid (20%), leucine (19%), alanine (12%), proline (10%), and reduced amounts of essential amino acids (lysine and tryptophan) [78]. Although the nutritional value of zein is significantly lower than the proteins of animal origin, it has some desirable properties that make it a promising ingredient for various applications. Zein is often used in food and industrial applications due to its unique properties, such as its high hydrophobicity and film-forming ability. Furthermore, zein is considered to be a safe and non-toxic ingredient for human consumption [8,125]. Protein oleogels were obtained using the emulsion template method by Wang et al. [130] with the aggregation of zein, which gelated different oils (olive oil, corn oil, and linseed oil). They showed that zein-stabilized oleogels have thermo-reversible properties while the oil phase had no significant influence on the oleogels’ thermal decomposition and crystalline structure.

##### Gelatin

Gelatin is a type of protein that is derived from collagen, which is the main structural protein of the connective tissues of animals. Gelatin is a mixture of peptides and amino acids produced through the hydrolysis of collagen of animal origin [131]. Gelatin is soluble in hot water, and when it cools, it forms a gel that can trap water and other substances. The strength and elasticity of the gelatin gel can be influenced by factors such as the concentration of gelatin, the pH of the solution, and the presence of other substances such as sugars or salts. This property makes it useful as a thickener, stabilizer, and gelling agent in a variety of food products [124,132]. An interesting application of oleogels is that they can be a good matrix for lipophilic bioactive molecules. Kavimughil et al. [40] found that the oleogel system formulated with gelatin, gellan gum, and medium-chain triglyceride (MCT) oil can be a delivery system for synergistic bioactive compounds, curcumin, and resveratrol. Furthermore, they observed that the two nutraceuticals were protected under oral and gastric conditions.

#### 2.2.2. Polysaccharides

Polysaccharides are some of the most abundant macromolecular polymers in nature that can be obtained from different sources such as algae, plants, animals, and microorganisms [133]. Their diverse extraction sources make them a good choice for use in various industries, including food, medicine, cosmetics, energy, paper, textiles, and fibers [134]. The use of polysaccharides in food processing is an attractive research field due to their multiple advantages: they come from renewable sources and are non-toxic, inexpensive, and positively perceived by consumers [135]. Due to their diversity, polysaccharides possess different physicochemical properties affecting their functionality and usage in various food products. For example, hydrophilic polysaccharides can be successfully used for formulating emulsion-based foods, such as oleogels [136,137].

The most commonly used polysaccharides as oleogelators are presented below.

##### Agar

Agar is a polysaccharide extracted from the cell walls of some species of red seaweeds such as *Gracilaria*, *Gelidiaceae, Pterocladiella capillacea*, and *Pterocladia lucida* [138,139]. It is a heterogeneous mixture made up of two polymers, agarose and agaropectin. Agarose, the major fraction of agar (70%), is a linear polymer containing two monosaccharides: β-d-galactose and 3,6-anhydro-α-l-galactose, linked by glycosidic bonds. The galactose residues can be substituted with sulfate groups but only below 0.15% [140,141]. The agaropectin molecule (30% of agar fraction) contains the units of agarose but also D-glucuronic acid and a small amount of pyruvic acid. Furthermore, a fraction between 3% and 10% of 3,6-anhydro-α-l-galactose residues is substituted by sulfate, pyruvate, or gluconorate residues, affecting negatively their gelling properties [142]. Agar is insoluble in cold water but dissolves readily in boiling water. By cooling it below 35–45 °C, a firm gel is formed even at concentrations as low as 0.1%. Agar gel is thermoreversible and, at a temperature higher than 80–85 °C, becomes liquid again [141]. It is a Generally Recognized as Safe (GRAS) food additive (E number E402), used as a gelling and thickening agent for jams, jellies, milk products, salads, dressings, or bakery products. In the last few years, some articles reported on agar usage as an oil structurant. Kodela et al. [41] describe in their study the synthesis of novel bigels prepared by mixing different proportions of stearyl alcohol oleogel with agar hydrogel. Microscopic evaluation showed the formation of biphasic or bicontinuous bigels, depending on the ratio between the two phases. The structural distribution of the components also influences the mechanical, viscoelastic, and electrical properties of the bigel system.

##### Xanthan Gum

Xanthan gum is an extracellular heteropolysaccharide produced by the microorganism *Xanthomonas campestris* during aerobic fermentation processes [143]. The xanthan molecule consists of repeated D-glucose, D-mannose, and D-glucuronic acid units in a molecular ratio of 2:2:1 and different proportions of acetyl and pyruvyl residues [144,145]. Xanthan gum has good solubility both in cold and hot water. Dissolved in cold water, even at low concentrations, it produces high-viscosity solutions (from 100 to millions of mPa·s) [146] with a weak gel character, stability over a wide range of temperatures, and pH 3–10 [147,148]. Due to these properties, it is used in the food industry as a thickener or suspending agent in dressings, sauces, instant desserts, dairy and bakery products, and fruit juices. Xanthan gum is accepted as a safe food additive in Europe (E number E415), the USA, and many other countries. Like other polysaccharides, Xanthan gum was also tested to obtain oleogels. Patel et al. [149] showed that the addition of xanthan gum stabilizes the polymeric network of a sunflower oil and gelatin oleogel obtained by the emulsion method. They proved that xanthan gum and gelatin interact with each other through hydrophobic and electrostatic interaction, confer higher stability of the oleogel, and prevent oil separation. In another study, Vélez-Erazo et al. [136] obtained structured sunflower oil by the emulsion method by using a two-component system: pea protein as an emulsifier and different polysaccharides (xanthan gum, carrageenan, gum Arabic, pectin, gellan gum, locust bean gum, sodium alginate, and tara gum) as stabilizers. The results showed that the oleogel stabilized by xanthan gum was one of the suitable systems in terms of the rheological behavior of emulsions, viscosity, and stability during storage.

##### Pectins

Pectins are structural polysaccharides present in the cell walls of most plants and have different functional properties. The term includes a group of substances differentiated by molecular weight, chemical configuration, and contents of neutral sugars [150]. Pectins are composed mainly of α-1,4-d-galacturonic acid residue linked by an alpha-1,4-glycosidic bond, with the following main structural domains: a linear chain of homogalacturonan and two types of highly branched rhamnogalacturonans (RGI and RG2). The pectin family also includes other classes of polysaccharides, such as xylogalacturonan, arabinan, galactan, arabinogalactan, and apiogalacturonan [51,151]. Usually, pectins are extracted from citrus fruit peels and albedo, apple pomace, banana peels, or sugar beet pulp [152,153]. Due to their lack of toxicity and their unique properties, pectins have valuable applications, especially in the food industry, where they are widely used as gelling agents, thickeners, emulsifiers, and stabilizers [154]. In the last years, polysaccharides have been successfully used as oleogelators to form structured oils. Luo et al. [61] obtained stable oleogels through the emulsion template approach using camellia oil, tea polyphenol-palmitate (2.5%), and citrus pectin at different concentrations (1.5, 2.5, 3.5, and 4.5%). They showed that the increase in citrus pectin concentration improved the stability and viscoelasticity of the emulsions and also the oil-binding capacity and gel strength of the oleogels. Furthermore, the addition of tea polyphenol conferred to oleogel good heat stability and high anti-oxidative activity. 

##### Chitin and Chitosan

Chitin is a long-chain polymer that is a primary component of the exoskeletons of arthropods such as crustaceans, some mollusks, and insects, and also of the cell walls of fungi. Chitin is composed of units of N-acety-D-lglucosamine, which are linked together by β-1,4 glycosidic bonds to form a strong and resistant material [155]. 

Due to its biodegradability and less-toxic nature, chitin is an attractive material for various applications, including the food industry, where it is used as a food additive or to obtain chitosan, a partially deacetylated counterpart of chitin [12]. Chitosan is used as a food preservative, antioxidant, thickener, stabilizer, and clarifying agent [156,157]. As good emulsifiers to stabilize heterogeneous oil-in-water systems, some application areas for chitin and chitosan include also oleogel formulation [158,159]. Baraki et al. [160] prepared sunflower oil oleogels structured by regenerated chitin by the emulsion templating method (approach). The resulting chitin-based oleogel showed high gel strength and good structural stability for a prolonged time. In another study, Brito et al. [55] obtained a stable oleogel using chitosan and canola oil with high thermostability and moderate oil-binding capacity. In the same work, chitosan was chemically modified by crosslinking with vanillin. The addition of vanillin (1%) improved the affinity of chitosan in oil and the mechanical properties of the chitosan-based oleogels.

##### Ethylcellulose

Ethylcellulose, is modified cellulose, which has one linear biopolymer built from anhydroglucose units, connected by glycosidic bonds. The hydrogen from the hydroxyl groups of these units is replaced with ethyl groups [161]. Being etherified, this biopolymer is also named cellulose ethyl ether, or ethyl ether of cellulose [162]. Ethylcellulose is a free-flowing, slightly hygroscopic white to off-white, odorless, and tasteless powder, with a density of 1.09–1.17 g/cm^3^, semicrystalline structure, and hydrophobic character [163,164]. It is a non-digestible and water-insoluble polymer [164]. Ethylcellulose is prepared from cotton linter or wood pulp by ethylation [164,165]. It is produced at different degrees of substitution values (DS) of ethoxyl groups. DS is one important parameter that quantifies the average number of derivatized hydroxyls, and it is used for the characterization of the product (e.g. solubility or average molecular weight) [164]. Thus, ethylcelluloses with DS 1–1.5 are soluble in water, while those with DS 2.4–2.5 are soluble in organic solvents [166]. It is a positive correlation between the DS, average MW, chain length, and viscosity of the polymer [58]. Several types of ethylcelluloses with average molecular weights in the range of 65,000–215,000 Da are sold, being characterized by the manufacturers by the viscosity value [167]. Ethylcellulose is accepted for use as a food additive. In the USA, it has a GRAS grade, and the EU included it in the official list of additives with E number 462. The hydrophobic nature and semi-crystalline structure of ethylcellulose explain its use as a gelling agent of plant oils by direct dispersion [92,161,168,169].

## 3. Food-Grade Oleogels: Analysis and Characterization

**Figure 2:**
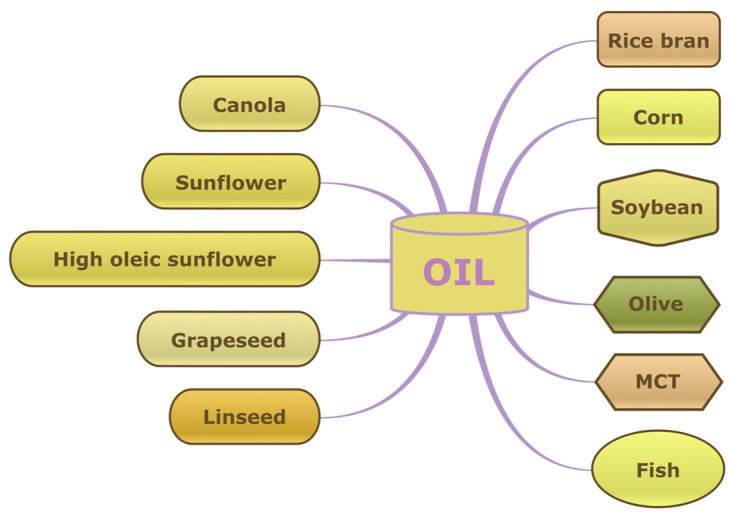
Different oils used for the preparation of food-grade oleogels.

Different oils or liquid hydrocarbons can be used as the base material for oleogels, depending on the desired properties and their further application. Some common oils used for oleogel formulations are presented in Figure 2.

The choice of oil for oleogel formulations depends on the desired characteristics of the final product, such as the desired texture, viscosity, stability, and functionality. When selecting the appropriate oil for oleogel formulations, the deciding factors are the cost, availability, regulatory requirements, and intended application, as well as a careful evaluation of the compatibility and stability of the chosen oil with other ingredients and additives in the formulation to ensure a stable and effective oleogel product.

**Figure 3:**
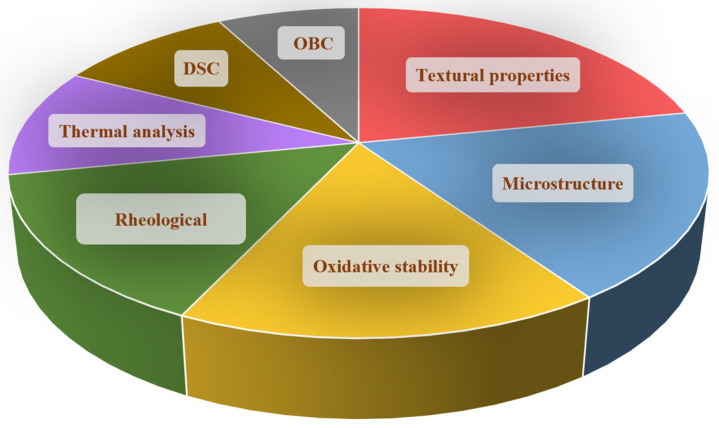
The most used methods of analysis for food-grade oleogels.

The analysis and characterization of oleogels involve a multi-faceted approach that includes compositional, structural, rheological, thermal, and microstructural analysis. These techniques provide insights into the properties, behavior, and performance of oleogels and can be used to optimize their formulation, processing, and utilization in various applications, including food, pharmaceuticals, and personal care products. Considering the food-grade oleogels analysis, laboratory studies should further consider comprehensive characterization referring to their sensory properties, shelf life, nutritional composition, processing behavior, compatibility, and microbiological safety. Figure 3 presents the most frequently used analysis methods in the case of edible oleogels. For this purpose, several databases were searched for the word “oleogel” and the considered analysis method. Thereafter, the graph was constructed considering the number of obtained hits for each search result.

### 3.1. Microstructure

The characterization of the structured lipidic systems in terms of their structure and interactions between the building blocks is a tremendous chore. Recent literature shows that some of the hypotheses on the microstructure of oleogels [24,66,88,95,170] and binary gels [171,172] can be considered rigorously defined. For the examination of the oleogel microstructure, the most often used method is polarized light microscopy due to the birefringence of the fat crystals. Although it is a very often used method for the identification of the formation of networks in oleogels, the inconvenience lies in micrometric measurements.

**Figure 4:**
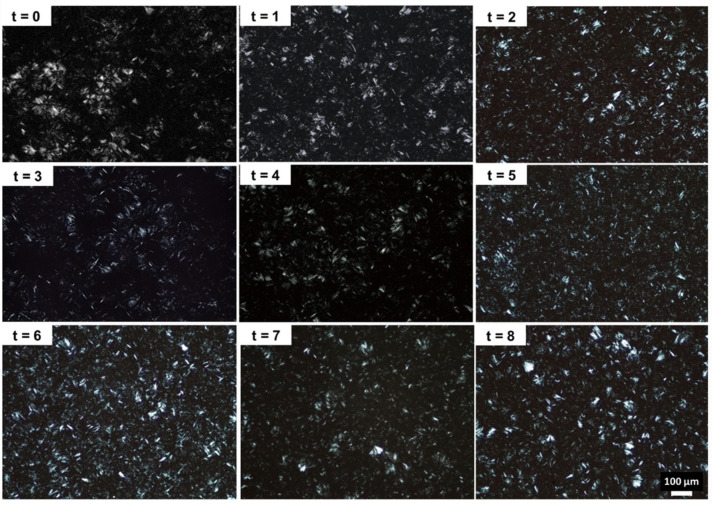
Polarized light microscopy (PLM) images of the monoglyceride oleogels stored at 5 °C over different storage times (t = weeks). PLM images were taken at 25× magnification. Taken from [177] with 2023 Copyright Clearance Center, Inc., license.

Table 2 presents the microstructure of several prepared food-grade oleogels, and it can be observed that waxes are often used and are efficient as crystalline oleogelators. However, it has to be pointed out that their oleogelation behavior is influenced by their surface area, the presence of solvents and their interaction with these, solvent polarity, oil components, oxidation reactions, etc. [111,161]. The registered photographs of the oleogels formulated with olive oil and monoglycerides show that the liquid phase of olive oil has a dark appearance, while the monoglycerides are identified as bright needle-like crystals [173]. The darkness is because olive oil is optically isotropic. Monoglycerides, as oleogelators, can grow over time and lead to the formation of multi-branched centers that might have different morphologies that will influence the mechanical, textural, and macroscopic properties of the formed oleogel [174,175,176]. Figure 4 presents the crystal size distribution in a monoglyceride oleogel stored for 8 weeks [177]. 

In general, for all of the considered systems presented in Table 1, the structure of the formed crystals is considerably influenced by the type of oleogelator and its purity, the used oil, and the conditions for the preparation of the oleogel, such as temperature and/or cooling rate, the presence of other additives, and shear application [8].

### 3.2. Rheological Properties

**Figure 5:**
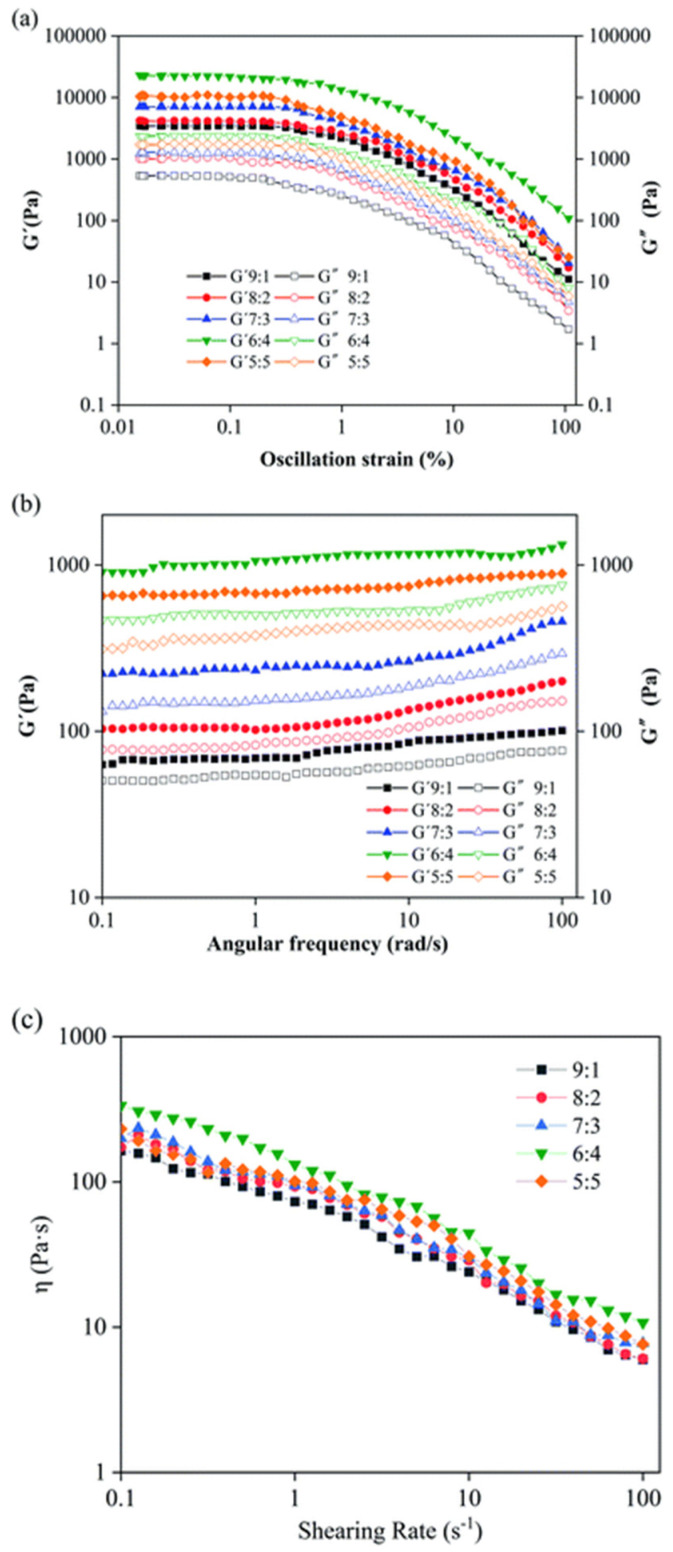
Rheological behavior of oleogels with different oil-to-water ratios. (**a**) Amplitude sweep, (**b**) frequency sweep, and (**c**) flow measurement curves. Taken from [196] with 2023 Copyright Clearance Center, Inc., license.

Rheological measurements can provide important insights into the properties and behavior of oleogels, which are important for numerous applications, especially for the food industry for the design of healthier and sustainable food products [195]. Moreover, the results from rheological measurements help in understanding the physical properties of food-grade oleogels and in optimizing their formulation. The oleogels’ rheological behavior can be investigated by employing oscillatory shear rheology and steady shear rheology. The first method is based on the application of sinusoidal stress or strain to the oleogel and measuring the resulting deformation response. This can provide information on the structure and mechanical properties of the oleogel, such as its storage modulus (elasticity) and loss modulus (viscosity). The steady shear rheology uses a constant stress or strain on the oleogel and registration of the resulting flow behavior that will contribute to the understanding of the viscosity and shear-thinning behavior of the oleogel. It has to be pointed out that the inconvenience of the rheological tests that are macroscopically performed is that they damage the samples. Figure 5 presents the effect of water on the rheological behavior of an oleogel [196]. 

Colloidal gels are made up of small, dispersed particles that interact with each other to create a network. The properties of a gel are largely determined by the strength of these interactions and how large and how numerous the dispersed particles are. It is difficult to control the aggregation of these particles, but this is important for a variety of applications, such as food, personal care products, pharmaceuticals, paints, or ink. In nonpolar solvents, the interactions between the particles are mainly led by steric and hydrogen bonding. When mineral oil and carbon black particles are combined, they form networks at a specific concentration of interaction energy [20,197]. This space-spanning network is endowed with elastic qualities as a result of hydrogen bond formation between individual particles. Adding a small amount of a secondary solvent can control the interaction between colloidal particles, resulting in fluid suspensions that can become elastic gels with a high yield stress. Wet granular media, where a wetting fluid increases inter-particle strength, are widely recognized. Wetting processes cause a liquid “bridge” between two particles to develop. Wetting and non-wetting fluids may create pendular or capillary gels, respectively. This method may change oil-based suspension viscosity in food applications [198]. The selection of a gelator is not easy to predict, as the final oleogel structure depends on complex interactions between network structure and oil chemical characteristics. Rheological analysis results of oleogels suggest that different types of oleogelators and oils can be used to find the best combination to apply in food-grade applications [199,200,201,202,203,204,205,206,207]. In recent scientific articles, different types of oleogels are analyzed by the rheological method to identify their properties (Table 3).

### 3.3. Oxidative Stability

Most of the preparation methods of oleogels are based on the mixing of an organogelator with an oil under continuous stirring and at high temperatures. Unfortunately, the high temperatures have a negative impact on the oxidation of the used oils. Therefore, one important consideration when using oleogels in food applications is their oxidative stability. Oxidative stability refers to the ability of a system to resist degradation due to the reaction of oxygen with the components that are in the system. When oils are exposed to oxygen, they can undergo a series of chemical reactions that lead to the formation of free radicals and other reactive species that can cause off-flavors, odors, and potential health concerns.

**Figure 6:**
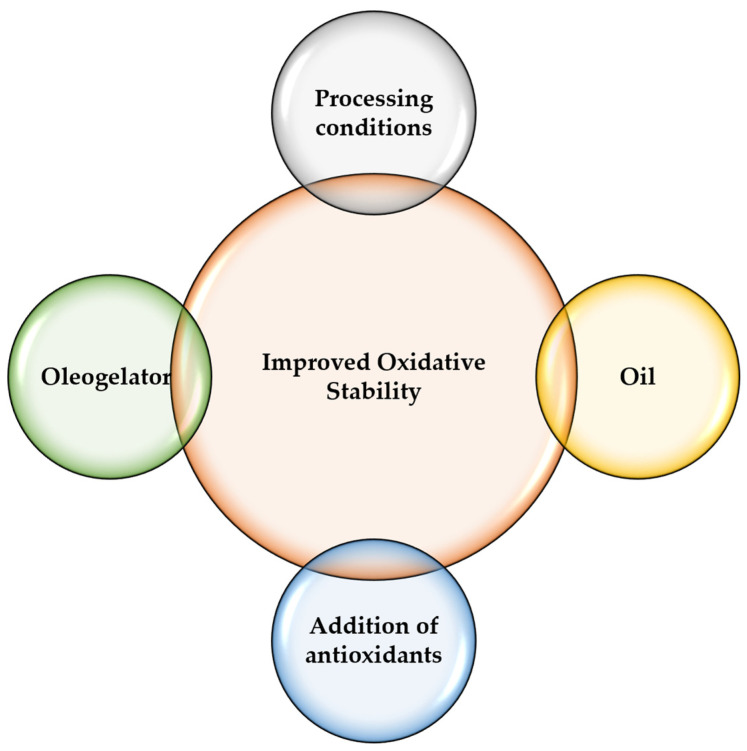
Factors affecting oxidative stability.

The oxidative stability of oleogels is influenced by several factors, including the type and amount of the structuring agent used, the type of oil used, and the processing conditions (Figure 6). 

For example, the use of unsaturated oils, such as soybean or sunflower oil, can lead to lower oxidative stability due to the presence of double bonds that are susceptible to oxidation. In contrast, the use of saturated oils, such as coconut or palm oil, can lead to higher oxidative stability. In a study published by Yilmaz et al., it was shown that oleogels formulated with beeswax and hazelnut oil showed higher peroxide values when kept at room temperature in comparison to those stored in a refrigerator [212]. Moreover, in the case of ethylcellulose, an often used organogelator, it was shown that its concentration and temperature and time of treatment play an important role in oxidative stability [162]. To improve the oxidative stability of oleogels, various approaches can be used, such as the addition of antioxidants or the use of processing conditions that minimize exposure to oxygen. Additionally, the choice of a structuring agent can have a significant impact on the oxidative stability of oleogels (Table 4). For example, waxes with high melting points tend to provide better oxidative stability than those with lower melting points. Overall, the oxidative stability of oleogels is an important consideration when using these systems in food applications, and careful selection of ingredients and processing conditions can help to improve this characteristic.

### 3.4. Textural Properties

The texture properties of a polycrystalline material refer to the distribution of crystallographic orientations in the sample. If the crystallographic orientations are not random but have some preferred orientation, the texture is weak, moderate, or strong. 

The optimal textural properties of an edible oleogel depend on the application and desired sensory experience. For example, an oleogel used as a spread or filling may require a smooth and spreadable texture with low adhesiveness, while an oleogel used in baking or frying may require a firmer texture with a higher melting point.

Overall, the textural properties of edible oleogels can be tailored by carefully selecting the type and amount of structuring agents and controlling the processing conditions to achieve the desired functionality and sensory attributes [161]. 

To determine texture properties, different texture equipment can be used, for example, texture analyzers and X-ray diffraction, and microscopes are used for qualitative analysis. 

The main purpose of oleogel production is to replace solid fats in food products. To do this, oleogels need to have the same texture and stability as fat molecules. Oleogelators are essential for creating products with the desired texture, and new trends in the field of oleogelation involve combining these technologies to get the best results [215].

**Figure 7:**
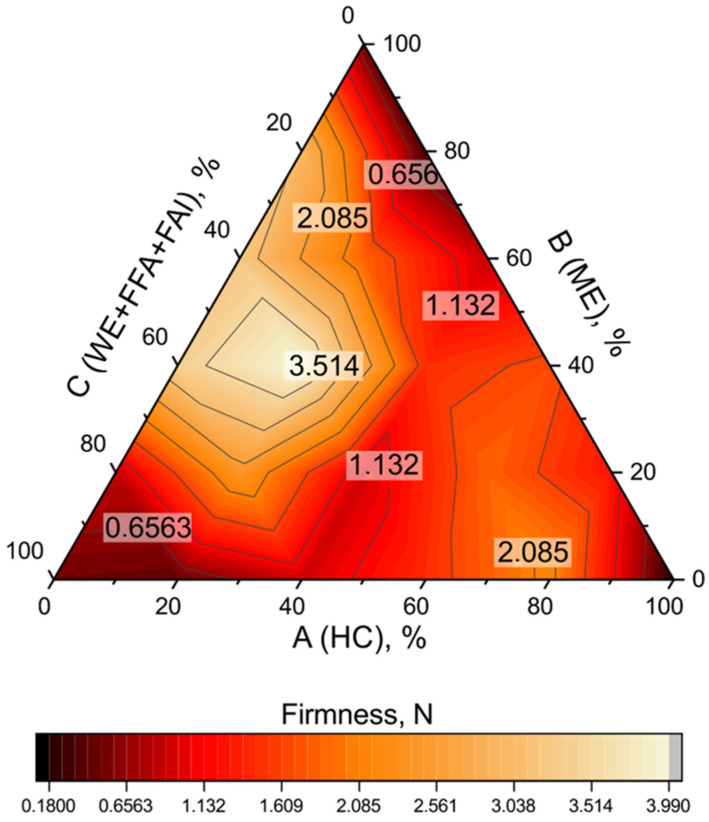
Firmness of oleogels structured with beeswax fractions. A, hydrocarbons > 99%; B, wax monoesters > 95%; C, 66% wax di- and triesters, >29% free fatty acids, and <5% free fatty alcohols. Taken from [71] with 2023 Copyright Clearance Center, Inc., license.

Oleogel characterization is important to create oleogels with better structuring capability, and the textural analysis determines the hardness, firmness, resistance, adhesiveness, cohesiveness, gumminess, and stickiness of gel stored under different conditions to choose the best oleogel that can replace the fatty acids for food product applications [215]. Sarkisyan et al. showed in their recent study the importance of an optimal combination of the fractions for obtaining oleogels with the desired firmness (Figure 7).

Recent scientific studies have shown that oleogels can be effectively used in food applications. The textural analysis of oleogels shows that different types of oleogelators and oils can be used to create the best texture for food-grade applications. For example, in Table 5 are shown different oleogelators and oils used to form oleogels. 

## 4. Applicability of Oleogels in Food

**Figure 8:**
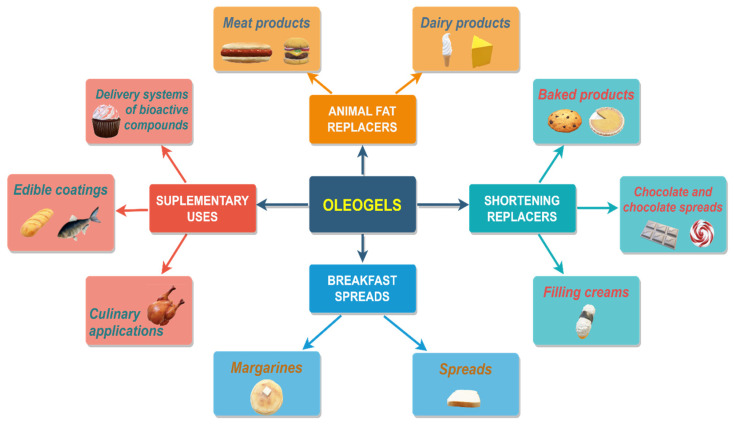
Application of oleogels in food in accordance with the intended purpose. Several studied and discussed products.

The literature review shows that oleogels have a wide range of potential applications in the food industry, including fat replacement, texture modification, shelf-life extension, nutrient delivery, reduced-calorie foods, clean-label and natural foods, and functional foods. Further research and development in this area are expected to unlock more opportunities for utilizing oleogels in food formulations. Figure 8 presents the application of oleogels in foodstuffs by grouping them according to their intended purpose in the design of novel products.

### 4.1. Oleogels Used as Animal Fat Replacers

#### 4.1.1. Meat Products

As a result of worldwide demand, there is now a real interest in research and industry to produce healthier meat products with a low saturated fat content and an improved fatty acid profile. Vegetable oils, rich in polyunsaturated fats, possess a liquid consistency that can lead to oil drain or undesirable texture if incorporated directly into foods in the technological process [169]. However, their advantage is the ability to be structured by edible oleogelators to obtain a solid-like structure suitable for incorporation as animal fat replacers in meat products, such as burgers, sausages, patés, patties, etc. [216]. 

The application of oleogels in meat products has been extensively studied in the past decade. A wide range of meat products has been investigated in terms of various oleogel incorporation and the results published in recent scientific literature: frankfurters and sausages [31,36,45,50,217,218,219], burgers [54,220,221,222], meat batters [223], patties [32,224], and patés [220,225]. 

Our research group has recently published an extensive overview on the use of oleogels in meat products as a technological solution for their nutritional improvement. The latest research and results on this topic are described in detail, highlighting the positive as well as the undesirable effects of formulating meat products with some newly developed oleogels. A relatively wide range of oils has been shown to be suitable for incorporation into meat products, such as soybean and high-oleic soybean oils, sunflower and high-oleic sunflower oils, olive oil, canola oil, rapeseed oil, linseed oil, sesame oil, rice bran oil, flaxseed oil, and mixtures. For their structuring, different oleogelators and obtaining methods were investigated. Thus, when the direct dispersion method was applied, oleogelators such as beeswax (BW), carnauba wax (CW), rice bran wax (RBW), sunflower wax (SW), ethylcellulose (EC), monoglycerides (MG), glycerol monostearate (GM), γ-oryzanol, β-sitosterol, and mixtures were dispersed, while hydroxypropyl methylcellulose (HPMC) was used when the indirect structuring method was applied. Fat replacement in meat products with the investigated oleogels also ranged from partial to total substitution. As an overall conclusion, although an improved fatty acid profile was obtained by the incorporation of oleogels into meat products, several negative effects of their use in terms of sensory and technical parameters were also observed. Therefore, further research in this area is needed to enhance sensory acceptance by consumers and to improve the technical functionality of oleogel-formulated products [169]. 

The design of oleogels should also consider investigating GRAS oleogelators and oil oleogelator formulation in terms of compatibility with meat products, as well as the addition of natural antioxidants to improve their oxidative stability and extend shelf life [226]. 

#### 4.1.2. Dairy Products

##### Ice Cream

One of the most studied dairy products in terms of oleogel incorporation is ice cream. Using oleogels as milk cream replacers in ice creams has resulted in lower saturated fat and healthier products. In addition, oleogels are innovative and promising solutions as ingredients in ice cream to achieve insignificant textural and rheological differences compared to conventional products, such as viscosity, hardness, and elasticity [227].

Maria Eletta Moriano et al. [228] evaluated the effects of milk cream substitution with sunflower oil oleogel on the properties of artisanal ice cream. The oil structuring was produced with a mixture of gelators, as follows: phytosterol complex concentrate:g-oryzanol in a ratio of 40:60 wt%. In terms of quality characteristics, comparable with traditional ice creams and even superior results were registered for the overrun and melting start time when an oleogel containing 12 g gelators/100 g oil was used. Moreover, the novel products were declared suitable for lowering blood cholesterol levels due to the presence of phytosterols in the composition [228]. 

Another recent study revealed the fact that carnauba wax oleogel, obtained on a soybean oil basis, can constitute an efficient replacer of saturated fat and a suitable ingredient in ice cream. Thus, soybean oil oleogel with 6% carnauba wax was used in the ice cream formulation by replacing 50% and 100% of the milk cream traditionally used as an ingredient. The results showed, in both substitution cases, a reduction in the melting rate of ice cream but also negative overrun aspects. However, after performing a sensory evaluation, the 50% oleogel formulation proved a similar level of acceptance with the control sample, with no significant differences in sensory characteristics [29]. 

An interesting study was performed by Sybilla Nazarewicz et al. [229], who presented opportunities for using tomato seed oil as a fat alternative in ice cream formulation, due to its excellent nutritional properties in terms of highly unsaturated fatty acid content. Moreover, an HPMC (hydroxypropyl methylcellulose) oleogel consisting of 60% cold-extracted tomato seed oil and 40% aqueous phase (2% HPMC solution) exhibited relatively high stability, with an impact on ice cream properties. Ice cream samples with different proportions of oleogel (5%, 6%, 7%) were studied in terms of several properties, such as fat, carbohydrate, and freezable water contents, which increased as the amount of oleogel became higher, and viscosity, hardness, and overrun, which decreased under the same conditions. The authors also analyzed the changes that appeared in the physicochemical properties of oleogel-formulated ice creams when ultrasound pasteurization was used instead of conventional pasteurization. The results showed that ultrasound technology, which uses a lower temperature, induced superior hardness and freezable water content to the products but inferior viscosity and overrun fat and carbohydrate contents. The authors concluded that this procedure can constitute a suitable alternative to classical pasteurization, with a similar effect, but further studies regarding the interaction of the time–temperature–ultrasound amplitude working parameters on the properties of oleogel-formulated ice creams are still needed [229].

##### Vegan Creams

There is also a real interest in obtaining vegan creams as an alternative to conventional dairy creams. In this regard, one recent research study tested the suitability of making vegan soy creams based on an organic soy drink, taking oleogel as an ingredient. Three different cream samples were analyzed, in which the fat phase consisted of milk fat (clarified butter), palm oil, and the oleogel obtained by structuring a mixture of rapeseed oil—linseed oil with 3% *w*/*w* candelilla wax. The designed oleogel showed better physical stability than milk fat, while the oxidative damage of the oil contained in the oleogel was limited. The vegan cream containing oleogel proved more resistant than the cream containing palm oil, making oleogel suitable as a substitute for conventional solid fats. The overall conclusion of this research indicates the possibility of achieving a vegan cream with similar physical characteristics to commercial UHT dairy cream [230]. 

##### Imitation Cheese

Currently, there is a tendency of formulating dairy-free imitation cheese, which is more and more used as an ingredient in foods. Its health benefits can be improved by reducing the fat amount in the composition. A study that aimed to produce an imitation cheese with a lower saturated fat content was conducted by Kyungwon Moon et al. [49], who studied the influence of palm oil substitution with oleogels on a dairy-free imitation cheese to achieve a lower level of saturated fat in the final product. Oleogels were prepared by structuring canola oil with different amounts of carnauba wax (3%, 6%, 9%) and then used as palm oil substitutes in the composition. The results showed that the level of saturated fats in the imitation cheese formulated with oleogels was significantly reduced from 45.7% to 5.2% and importantly enriched in unsaturated fats, with a saturated: unsaturated fat ratio decreasing from 0.84 to 0.06. Moreover, the textural properties of oleogel-formulated products were enhanced in terms of elasticity, hardness, and cohesion [49]. 

### 4.2. Oleogels Used as Shortening Replacers

#### 4.2.1. Baked Products

In baked products, fats play an important role in terms of their sensory properties such as texture, color, taste, and flavor, whose interaction influences the overall consumer acceptance. Bakery and pastry products require large quantities and different types of fat as ingredients, especially shortenings and baking margarine or butter, to obtain a wide range of bakery products such as cakes, pastries, cookies, biscuits, bread, etc. Because of the high consumption of these types of foods, it is of utmost importance to find solutions for the development of fat-reduced baked products. This is a real challenge today, as conventional fats confer certain textural, rheological, and sensory properties that need to be preserved even when using fat substitutes. Structured oils, such as oleogels, are alternatives to maintain the solid-like properties of products, and they also possess a healthier fatty acid profile. Thus, in recent years, researchers in this field have paid particular attention to exploiting oleogels to obtain nutritionally improved and low-fat bakery products [48,231,232].

Baked products have been largely explored in terms of incorporating oleogels, aiming to replace the hydrogenated shortenings or animal fats, reduce the trans and saturated fats, and improve their nutritional profile. Quite a few studies have been published, with a focus on cakes [54,233,234,235,236], cookies [204,237,238], biscuits [239,240], buns [241], and muffins [1]. Sunflower, rice bran, beeswax, candelilla, shellac, and carnauba waxes were the prevalent oleogelators used for structuring mainly vegetable oils such as olive, soybean, canola, sunflower, rapeseed, and hazelnut. In addition, only a few studies have designed oleogels for baked products using hydroxypropyl methylcellulose, methylcellulose, ethylcellulose, and monoglycerides as gelation agents.

An extremely interesting and unique study was conducted by Doyoung Kim et al. [237], who obtained an oil rich in unsaturated fatty acids from the larvae of an edible insect, *Tenebrio molitor*, which stands out as a possible protein supplement or future food material. So far, few studies have been carried out on the food use of this type of oil, extracted at a low temperature by compression. After investigating the antioxidant activity and fatty acid composition, the extracted oil was used to obtain oleogels with different waxes, such as candelilla, carnauba, and beeswax. Through oleogelling, the oxidative stability of the oil during storage was improved. By replacing the conventional shortening in the cookies with the designed oleogels, desirable properties in terms of spread factor and texture were obtained. The hardness recorded in the oleogel cookies with beeswax and candelilla wax was lower compared to that of the classical cookies, while the spread factor and textural attributes in the samples with carnauba wax oleogel were insignificantly different. As a general conclusion, the use of *Tenebrio molitor* larvae oil in the form of oleogels proved to be a suitable alternative for the replacement of solid saturated fats in cookies and for their nutritional enrichment by improving the fatty acid profile, but further studies are needed regarding the incorporation of these types of oleogels in other food systems [237].

#### 4.2.2. Chocolate and Chocolate Spreads

Several published studies on the application of oleogels in chocolate and chocolate spreads have explored the development of heat-resistant chocolate, the replacement of oil binder in chocolate paste, the substitution of palm oil in the fillings or cocoa butter in composition, and the control of fat bloom induced by oil migration. A reduction in saturated fats in chocolate and chocolate spreads and an improvement in their fatty acid profile have been reported in a few recent studies that proceeded to the incorporation of oleogels as replacements for conventional fats [242,243].

M. Espert et al. [244] developed an oleogel based on sunflower oil and hydroxypropyl methylcellulose (HPMC), obtained by the emulsion template method and subsequently partially replacing the cocoa butter in the chocolate composition. Various concentrations of HPMC (0.5%, 1%, 1.5%, and 2%) were investigated, while the cocoa butter replaced in chocolate was 50%. The results showed that HPMC-structured sunflower oil is a suitable alternative to cocoa butter in chocolate, while the optimal concentration of HPMC within the oleogel was considered to be 0.5% and 1%. Thus, the sensory attributes such as texture and flavor were very similar to the control chocolate produced with cocoa butter [244].

Likewise, to reduce saturated fats and produce healthier chocolate with an improved fatty acid profile, Behraad Tirgarian et al. [245] designed a low-fat chocolate spread using water-in-oleogel emulsions. Corn oil was used as the base and glycerol monostearate (20% wt%) as the oleogelator, while the emulsion was prepared in various weight ratios, as follows: 0:100, 45:55, 50:50, and 55:45 (water:oleogel). Physical, rheological, and sensory characteristics of the developed chocolate spreads with 44% water-in-oleogel emulsion content were analyzed. The chocolate spread sample with 45% oleogel water replacement was found to be the most similar to traditional cocoa butter in terms of mouthfeel. Sensory scores and acceptability were comparable to the control. Some textural properties, such as firmness and spreadability, still require further testing for optimization, as well as the water activity that needs to be reduced for inhibiting microbial growth [245]. 

#### 4.2.3. Filling Creams

Some applications of oleogels as shortening substitutes in filling creams can be noticed in a few recent studies, as follows: Minhyo Kim et al. [246] tested the efficiency of a binary mixture of oleogelators in obtaining oleogels suitable for low-saturated fat filling creams. In this regard, candelilla wax (CDW) and glycerol monostearate (GMS) were used as gelation agents, in varied proportions, to achieve canola-oil-based oleogels. The CDW: GMS 60:40 ratio provided an oleogel with convenient properties in terms of hardness and melting point for the intended purpose. The oleogel-containing filling creams were found to possess similar textural properties as the shortening-containing ones, with direct consequences on the avoidance of oil mitigation in products. At the same time, the saturated fat content in the creams designed with oleogel significantly decreased from 36.2% to 10.3%. The authors recommend the binary combination of gelators in achieving suitable oleogels for filling creams to limit the disadvantages of the usual utilization of a single oleogelator [246]. 

Providing spreadable oleogels as ingredients for various foods is a challenge, to obtain suitable products in accordance with the aimed purpose. Three spreadable oleogels with functional properties were developed by Heba H. Salama et al. [247] for further use as a healthy and nutritious ingredient in candy gummies. A basic oleogel was obtained by structuring canola oil with 10% stearic acid as an oleogelator and three other spreadable oleogels with functional properties by enriching it with some nutritive polymers such as casein, starch, whey protein, and maltodextrin. Doum fruit was a source of polyphenols and flavonoids for one oleogel sample, while carrot juice enhanced the other oleogel’s content with β-carotene. Nutritionally improved candy gummies were obtained on the base of developed spreadable oleogels and showed high acceptance in sensory evaluation, constituting a functional product with a high content of unsaturated fats and antioxidants [247]. 

### 4.3. Oleogels Used in Breakfast Spread Developing

#### Spreads and Margarines

In the past decade, several studies have been conducted on the use of oleogels in the formulation of breakfast spreads. Thus, Patel et al. [232] evaluated shellac oleogels in various edible applications, including spreads, meaning structured water-in-oil emulsions with 60% wt% water and no emulsifier due to the integration of shellac oleogels as the continuous oil phase. They showed good stability for more than four months, and the authors concluded that shellac oleogels are suitable for such food systems [232]. The use of shellac wax as an oleogelator was recently studied by Andreea Pușcaș et al. [248] for identifying the optimal formulation for a low-fat spread designed with sunflower oil. Rheological, textural, colorimetric, and stability determinations were performed on shellac wax-based water-in-oil emulsion oleogels with various water proportions (20–60% *w*/*w*) and different shellac wax concentrations ranging from 3% to 7% *w*/*w*. The results provided some conclusions, including that adequate functionality occurs when the water content is up to 40–60% *w*/*w* and the properties are similar to those of margarine. Stable emulsions can be obtained even with 3% *w*/*w* shellac wax when the water content is up to 60% *w*/*w*. The formula provided by the multidimensional statistical analysis for shellac wax emulsion oleogel for the development of an optimal low-fat alternative margarine was found to be 4.29% *w*/*w* shellac wax and 24.13% *w*/*w* water [248]. 

A microbial oil wax-based oleogel was developed by Aikaterini Papadak et al. [183]. As oleogelators were used bio-based wax esters derived from microbial oil produced by the oleaginous yeast *Rhodosporidium toruloides* by enzymatic conversion with lipases. The obtained cetyl ester wax was then used for structuring olive oil to develop an oleogel that was further analyzed in terms of rheological and thermal properties. The results led to the conclusion that this oleogel is proper for application in spreadable fat products, due to its low melting behavior and firmness which was found to be similar to the breakfast margarines on the market [183]. 

A great potential of oleogels is to be a suitable and feasible alternative to commercial margarines obtained by the traditional hydrogenation method, due to their low content of saturated fats and lack of trans fatty acids. In this respect, one recent study has evaluated oleogel-based margarines. Xiuhang Chai et al. [249] first developed an oleogel based on high-oleic sunflower oil with beeswax incorporated as an oleogelator in various concentrations (4%, 6%, 8%, 10% wt5), which was subsequently used for preparing the oil phase which was then mixed with the water phase in a ratio of 80:20 (*w/w*). Rheological and textural properties and microscopic morphology were analyzed and compared with those of classical margarine. The hardness of oleogel-based margarine increased with beeswax concentration, but negative mouth melting was observed at its highest concentration. An important observation revealed that a fast-cooling process imparted higher hardness and superior spreadability to the products compared with the slow-cooling method. In addition, enhanced physical properties were achieved when using a binary blend of oleogelators, meaning China lacquer wax (ZLW) and beeswax (BW) 50:50. The overall conclusion of this research was that the best replacement potential for conventional margarine, in terms of appearance, mouth melting, and mechanical properties, was exhibited by oleogel-based margarine when 5% BW50:ZLW50 was used for oil structuring and the fast-cooling method was applied [249]. 

### 4.4. Supplementary Uses of Oleogels in Foods

#### 4.4.1. Edible Coatings

Yi Chen et al. [250] studied the influence of treating bread crust with several edible oleogel-based coatings, applied to minimize its water loss during storage at ambient temperature. A comparative study was carried out in terms of water migration and bread staling when various treatments were applied to the bread crust, such as gum-based, protein-based, starch-based, ethylcellulose oleogel, wax-based oleogel, and HPMC (hydroxypropyl methylcellulose) oleogel. In all cases, it could be noticed that weight loss and crumb firmness were significantly lower compared to untreated bread. However, the sunflower-oil–oleogel coatings with 20% candelilla or 20% beeswax proved to be the most efficient, due to their low water affinity and permeability to water vapors. Effective results were also obtained when HPMC oleogel coating was applied, probably due to its high oil concentration and very compact structure. The overall conclusion is that the named wax-based oleogels are suitable for use as a water barrier in some other foods with high water content, while cellulose derivatives may constitute viable alternatives [250]. 

For extending shelf life and reducing packaging requirements, some edible coatings in the form of hydrogels (H), oleogels (O), and bigels (BG) were evaluated by Aikaterini Kanelaki et al. [39] as regards oxidation delay and microbial spoilage in fresh sardine fillets. In addition, one group of gel (H, O, and BG) coatings was enhanced with rosemary extract (RE) as an antioxidant agent. The reported results demonstrated that the use of gel coatings inhibited lipid oxidation, while the incorporation of RE significantly retarded oxidation processes in this fish product. However, the addition of RE into the gel coatings did not show any changes in the growth of microorganisms during storage. The embedding of RE in the oleogel as part of the bigel proved to be the most suitable coating option for sardine fillets in terms of quality maintenance under refrigeration storage conditions and good potential in bioactive compound delivery [39]. 

#### 4.4.2. Delivery Systems of Bioactive Compounds

One of the most interesting applications of oleogels consists in the delivery of bioactive molecules, as they are studied to be efficient delivery systems for functional compounds in the human body. Although most studies focus on exploring the incorporation mechanism, behavior, and bioaccessibility of oil-soluble bioactive compounds, there are several studies that address also the integration of water-soluble compounds into such systems, such as emulgels or bigels. Thus, there is a real perspective in exploring lipophilic and hydrophilic functional molecules as components in oleogels and oleogel-based systems, in terms of their production, characterization, degradation mechanism, and absorption during digestion. Moreover, their further applicability in food products is also a challenge to develop innovative foods with additional functional properties [2,251]. 

Various functional compounds and their incorporation into oleogels and subsequently into various food products have been studied, and the results are disseminated in recent scientific literature. Sohui Jeong et al. [1] tested an antioxidant-fortified oleogel obtained by structuring sunflower oil with candelilla wax enriched with glycerol monostearate (GMS) at a ratio of 1:3 and adding β-carotene (0.02%) (OGB). The fortifying of candelilla wax with GMS led to improved rheological properties of oleogels, such as strength and viscoelasticity, while the addition of β-carotene as an antioxidant resulted in the highest oxidative stability of the designed olegogels. The volume and porosity of OGB-oleogel-formulated muffins that were further developed revealed insignificant differences compared to shortening traditional muffins but a harder texture and the highest oxidative stability [1]. 

Another antioxidant used in the formulation of oleogels is vitamin C, which was studied as an ingredient in margarine production by Xing Wang et al. [14]. Thus, a vitamin-C-loaded oleogel (VCOG) was developed on a base of corn oil, structured by using monoglyceride stearate as an oleogelator (12% *w*/*w*) and further incorporated into margarine as a replacer of its total lipid phase. As regards the oxidative stability of the oleogels, the registered peroxide and p-anisidine values in VCOG were significantly lower than in the oleogel without added vitamin C (*p* > 0.05). Four commercially available butters were used as controls to evaluate the physical and sensory properties of VCOG. The results reported the lowest levels of solid fats and trans fatty acids, as well as the lowest firmness in VCOG, while unsaturated fatty acid content and stickiness showed the highest levels. The overall impression, together with the scores for texture, appearance, taste, and some textural properties, such as elasticity, cohesiveness, and gumminess, registered similar values to some/all commercial butter (*p* > 0.05) [14].

The loading of oleogels with curcumin, a natural polyphenolic compound with biological properties, has been tested in several studies in terms of oral delivery and release rate. Anashwara Babu et al. [37] prepared and characterized new oleogels based on wheat germ oil with jasmine floral wax used as an oleogelator and curcumin loaded and analyzed its oral delivery and release by ex vivo intestinal permeation studies. The results indicated that the designed oleogels achieved the necessary characteristics to modulate curcumin delivery, with potential applications in food and pharmaceutical products [37]. Moreover, in this regard, Yuanyuan Xu et al. [43] developed walnut oil oleogels with different proportions of methylcellulose, prepared by the emulsion method, some of which were loaded with curcumin (0.5 wt%) to serve as delivery systems. Some polysaccharides, such as κ-carrageenan, sodium alginate, and xanthan gum were added as thickening agents. The digestive properties of the curcumin-loaded oleogels were evaluated by in vitro tests. The results showed that the highest encapsulation rate of curcumin (38.06%) was recorded in κ-carrageenan oleogel, which presented a higher inhibitory effect. As an overall conclusion, this study provided suitable systems for the delivery of lipophilic bioactive compounds, namely biopolymer-based walnut oil oleogels, and a new concept of the functional alternative to solid fats [43]. 

Few recent studies have also been conducted regarding the design of different food-grade oleogels as delivery systems for astaxanthin, an agent used as a nutritional supplement primarily due to the unusual antioxidant activity it possesses. The reported results are promising as regards the efficacy of oleogels as delivery materials of astaxanthin, by enhancing its stability and bioaccessibility, but further studies are needed for testing their performance in real food matrices [44,53].

#### 4.4.3. Culinary Applications

Oleogels have also been tested as a frying medium. For this purpose, the fat absorption behavior of several foods and some resulting sensory characteristics were evaluated. For example, Kelvin Adrah et al. [252] studied the oleogel (OG) obtained by structuring canola oil with carnauba wax (5%, 10% *w*/*w*), which was subsequently used for deep frying chicken breast for 3–4 min at 177.7 °C. Comparing the fat absorption of chicken breast samples fried in canola oil (15.10%) with the fat absorption of the samples fried in canola-oil-based oleogels, the results registered lower values: 8.53% for OG 5% and 9.15% for OG 10%. The protein and ash contents were not influenced by the frying medium, but the water content was significantly higher in samples fried in oleogel. However, differences were observed in the appearance of all crust-fried samples, such as pore size and fissures, as well as a color variation, probably not detectable by consumers. The textural properties of the samples were not negatively affected by the oleogel frying. Although the results of using oleogels for this purpose are promising, further sensory evaluation of the resulting products is recommended [252]. 

A traditional Indian snack, mathri, has also been studied in terms of deep frying in oleogel to reduce fat uptake and obtain a low-fat product. Thus, as a frying medium were used soybean-oil-based oleogels (OGs) structured with carnauba wax (5%, 10%, and 15%) and soybean refined oil (SO) as control. Fat absorption was lower in the samples deep-fried in oleogel by 27.7% for OG 5%, 22% for OG 10%, and 19.3% for OG 15% than in the control sample deep-fried in soybean oil. At the same time, moisture retention, color, and texture were better in the oleogel-fried samples, while crispiness was similar between the samples fried in OG 15% and the control. The overall conclusion of the study was that deep frying using oleogels in mathri resulted in reduced fat and calorie content, with an emphasis on health benefits. However, further studies are recommended to evaluate different formulations of oleogels in the context of frying conditions [253].

Some other applications of frying in oleogels have been reported by Buket Aydeniz-Guneser et al. [254], who tested sunflower-oil-based oleogel as an alternative medium for onion ring frying in refined sunflower oil, and Paramee Noonim et al. [9], who evaluated the effects of frying instant noodles fortified with salted duck egg white in palm-oil–carnauba wax oleogel compared with the same product traditionally deep-fried in palm oil. 

In the first study, sunflower oil was structured with 3% and 8% polyglycerol stearate and further used for deep frying onion rings, which were finally tested in terms of sensorial and textural properties, and the results were compared with those of the control samples. The recorded fat uptake was lower in onion rings fried in oleogels by about 33–37% than in control samples, which generated a reduction also in the energy value of the product with positive consequences in weight gain. Moreover, the textural profile and overall acceptability score were higher than in the control samples [9,254]. Paramee Noonim et al. [9] carried out their study by first elaborating on palm-oil-based oleogels with carnauba wax in different concentrations (5%, 10% *w*/*w*), some of which were processed by ultrasonication. These were subsequently tested as a frying medium for instant noodles fortified with salted duck egg white. The consequences of the frying process in various mediums on their physicochemical properties were evaluated and compared with the control. Significant positive effects on the physical and chemical properties of the product were reported when the palm oil oleogel with the highest concentration of carnauba wax and ultrasonicated was used. The cooking yield, cooking time, and textural profile (hardness, firmness, elasticity, tensile strength, and chewability) were improved, while the oil absorption was lowered by using oleogels. Better results were reported for the samples deep-fried in ultrasonication-assisted oleogels, in terms of surface appearance. Finally, the oxidative stability proved to be much higher in noodles fried in oleogels than in oil-fried samples. By corroborating all the results, the general conclusion was that the designed oleogels are suitable alternatives to conventional oil used for deep frying instant noodles fortified with salted duck egg white [9]. 

## 5. Conclusions and Future Perspectives

Oleogelators are the main components of the oleogelation process. Depending on their structure, concentration, and formulation conditions, the oleogelation mechanism will occur, and properties such as microstructure, oil-binding capacity, and sensorial, rheological, and textural properties will be influenced. Furthermore, the final properties of oleogels can be improved by mixing more oleogelators with synergistic effects or by using nongelling additives together with the oleogelator. However, the choice of a gelator cannot be easily predicted, since the final structure of the oleogel depends on complex interactions between the network structure and the chemical characteristics of the oil. 

Oleogels are a promising ingredient for the food industry due to their ability to create structured networks in liquid oils, leading to improved textural and functional properties in food products. However, there are several challenges in developing oleogels for food applications, including the finding of the structuring agents that are Generally Recognized as Safe (GRAS), ensuring that the final food products are safe for consumption without any health risks and exhibit economic viability, palatability, application versatility, and shelf-life stability. Despite these challenges, oleogels have shown great potential for various food applications, and ongoing research and development efforts are being made to unlock the full potential of oleogels in the food industry. 

The structuring of edible oils has registered considerable progress in recent years, and the interest in developing suitable oleogels for various products and application in real food matrices is constantly growing. Considering all the aspects discussed in this review, it is obvious that oleogels are a promising alternative to saturated fats in various investigated food products, and other potential foods should be further investigated in this regard. Particular attention should be paid to the sensory properties of oleogel-formulated products so that no significant changes compared to traditional products occur. Moreover, the palatability of the designed products, and therefore the overall consumer acceptance, should not be negatively influenced.

In conclusion, based on the results of several extensive and varied studies, oleogels have shown potential for usage in various food products, including bakery goods, breakfast spreads, margarines, chocolates, and some meat products, due to their ability to improve texture, reduce saturated fat content, enhance shelf stability, and offer flexibility in formulation. However, challenges such as cost considerations, processing challenges, limited applications, and potential sensory changes should be carefully considered when formulating products with oleogels. Further research and development efforts are needed to optimize oleogel formulations and expand their applications in the food industry.

## Figures and Tables

**Table 1:** The structure or composition of oleogelators.

Oleogelator	Structure/Composition
Low-molecular-weight oleogelators	
Fatty acids	
Stearic acid (Octadecanoic acid) n = 18	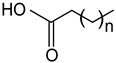
Esters of fatty acids	
Sorbitan monostearate	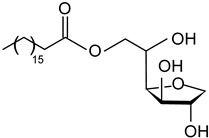
Waxes	
Beeswax	- Fatty acid esters (58–71%). Mono-, hydroxy mono-, and di-esters with a chain length of C24–C32. - Hydrocarbons (12–26%). Alkanes and alkenes with chain length of C27–C33. - Free fatty acids (9–15%). - Free fatty alcohols (1–6%). [68,69,70,71,72]
Carnauba wax	- Fatty acid esters (62–85%). - Free fatty acids (3–6%). - Free fatty alcohols (10–30%). [6,68,72]
Sunflower wax	- Fatty acid esters (over 95%). Chain length of C48–C84. - Free fatty acids (ca. 3%). - Free fatty alcohols (ca. 0.3%). - Hydrocarbons (ca. 0.17%). [68,70]
Rice bran wax	- Fatty acid esters (over 93%). Chain length of C44–C50. - Free fatty acids (ca. 1%). - Free fatty alcohols. [68]
Glycerolipids	
Monoglycerides	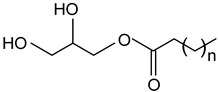
Glycerophospholipids	
Lecithins (Phosphatidylcholine)	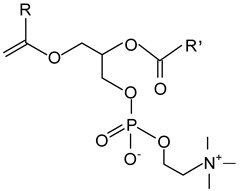
Sterol lipids	
Phytosterols β-sitosterol	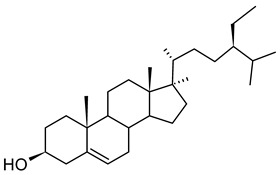
Phytosterol esters γ-oryzanol	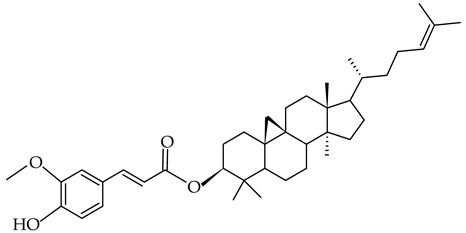
Sphingolipids	
Ceramides	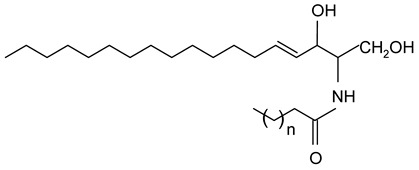
Fatty alcohols	
Stearyl alcohol (1-octadecanol) n = 18	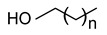
High-molecular-weight oleogelators	
Proteins	
Soy protein	- Soy proteins are complete proteins since they contain all of the essential amino acids. - Up to 90% of the total proteins are storage proteins represented by glycinin and β-conglycinin. - Glycinin (65–80% of total protein), classified as a legumin, is a hexamer with molecular mass between 300 and 380 kDa. - β-conglycinin (30–50% of total proteins), classified as a vicilin, is a trimer with molecular mass between 150 and 200 kDa. [69,73,74]
Whey protein	- The major whey proteins are β-lactoglobulin and α-lactalbumin. - β-lactoglobulin (50–55% of the whey protein) is a globular protein with molecular mass 18,277 kDa. - α-lactalbumin (20–25% of the whey protein) is a smaller globular protein with molecular mass 14,175 kDa. - Bovine serum albumin (5–10% of the whey protein) is a globular protein with molecular mass of 66.5 kDa. [75,76,77]
Zein	- Is a class of prolamin protein consisting of a mixture of three fractions including α-zein as major fraction (75–85%), β-zein, and γ-zein. - The amino acid profile of zein includes glutamic acid (20–26%), leucine (19–20%), alanine (10–12%), proline (10%), and reduced amounts of lysine and tryptophan. - Zein is a storage protein with molecular weight of 44 kDa. [78,79]
Gelatin	- Gelatin is a mixture of peptides and proteins resulted by partial hydrolysis of collagen. - The amino acid profile of gelatin consists of glycine (27–35%), proline (10–18%), alanine (8–11%), hydroxyproline (20–24%), arginine (8–9%), aspartic acid (6–7%), and glutamic acid(10–12%). [80,81]
Polysaccharides	
Agar	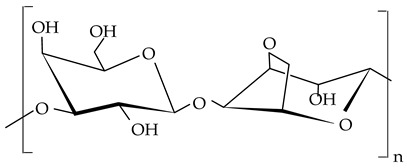
Xanthan gum	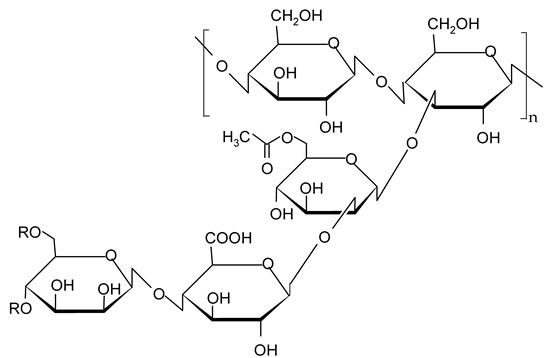
Pectins	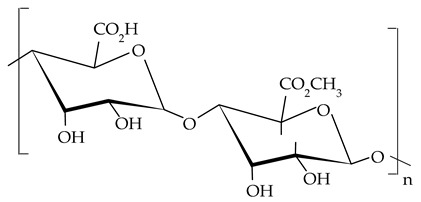
Chitin and chitosan	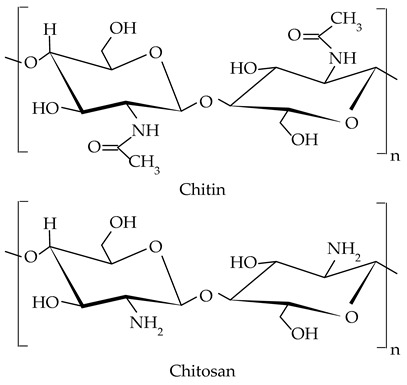
Ethylcellulose	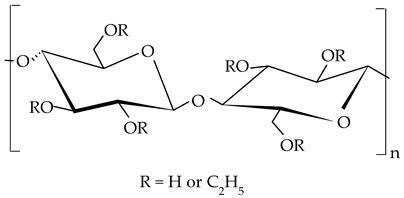

**Table 2:** Microstructural characteristics of oleogels.

Organogelator	Oil	Type of Microscopy	Results	Comments	Reference
Beeswax	Sunflower Flaxseed	Polarized light microscopy	Small number of small and rod-shaped crystals	Higher concentrations of beeswax contributed to formation of denser and larger crystals The type of oil did not have influence on the microstructure of the oleogel	[178]
Beeswax Beeswax hydrocarbons	Deodorized bleached sunflower	Polarized light microscopy	Needle-shaped crystals for oleogels prepared with beeswax and hexagonal crystals for beeswax-hydrocarbon-based oleogels	The cooling rate of the oleogels influences the size of the formed crystals	[88]
Beeswax	Virgin olive oil	Polarized light microscopy	Needle-like beeswax crystals	Preservation of the oleogels for 90 days at room temperature did not influence their structural stability	[179]
Beeswax	Sunflower oil	Polarized light microscopy	Needle-like crystals	The needle-like crystals are seen like this, but in reality, they have platelet-like shape due to the crystals perpendicular to the surface of the slides	[71]
Beeswax	Fish oil Sunflower oil Olive oil Linseed oil	Polarized light microscopy	Needle-like crystals, exception for linseed-oil-based oleogel where the crystals are mainly spherulitic in shape	The structure and the size of the crystal are related to the oil-binding capacity and textural performance	[180]
Candelilla wax Monoglycerides Hard fat	High-oleic sunflower	Polarized light microscopy	Spherulite-shaped crystals formed by radially oriented needle-like crystals	Addition of water changed the size and morphology of crystals	[181]
Carnauba wax	Refined soybean oil Refined peanut oil	Polarized light microscopy	Needle-like crystals	An increase in the carnauba wax content leads to the aggregation of the crystals	[29]
Ceramide III Soybean lecithin	Sunflower oil	Polarized light microscopy	Spherulite-like aggregates formed by radially oriented needle-like crystals	Addition of water to the system resulted in the formation of lamellar crystals due to the re-arrangement of ceramide and lecithin	[182]
Cetyl esters wax	Olive oil	Polarized light microscopy	Flake-like crystals	The cooling temperature influences the size of the crystals	[183]
Ethylcellulose Stearic acid Octadecanol Monoglyceride Beeswax Rice bran wax Sunflower wax Mixture of γ-oryzanol and β-sitosterol	Sunflower oil	Polarized light microscopy	Needle-like crystals In the case of rice bran wax oleogels, spherical crystals were observed, while for those with stearic acid and octadecanol, the crystals were platelet-like structures	Except for the mixture γ-oryzanol - β-sitosterol and monoglyceride-based oleogels, all the other oleogels did not show changes in their microstructure	[184]
Ethylcellulose Beeswax	Olive oil Linseed oil Fish oil	Polarized light microscopy	The ethylcellulose oleogels had polymeric structure with holes in which the oil is entrapped The beeswax oleogel has a needle-like structure	Needle-like crystals permit the entrapment of large amounts of oil	[89]
Hydroxypropyl methylcellulose with different concentrations of pectin	Medium-chain triglyceride oil	Optical microscopy	Small droplets	Addition of pectin contributes to the formation of smaller droplets	[185]
Monoglycerides	Olive oil	Polarized light microscopy	Large crystals	-	[173]
N^α^, N^ε^-diacyl-L-lysines	Linseed oil Soybean oil gel Corn germ oil Olive oil	TEM	Needle-like crystals that have different lengths dependent on the used gelator	The type of used oil does not influence the microstructure of the oleogels. This depends on the carbon-chain length of gelators	[34]
Rice bran wax	Soybean oil	Polarized light microscopy	Dendritic crystals	An increase in rice bran wax content leads to an increase in the crystal size	[186]
Rice bran wax Added low-melting-point fractions of cocoa butter	Corn oil	Polarized light microscopy	Needle-like crystals	An increase in the content of low-melting-point fractions of cocoa butter contributes to the formation of crystallites with the possibility to form a crystallization network	[187]
Sunflower wax Carnauba wax Candelilla wax Beeswax Berry wax Fruit wax	Refined high-oleic sunflower oil	Normal and polarized light microscopy	Rod-like crystals	The high content of wax esters contributes to the formation of rod-like morphology	[188]
Sunflower wax Beeswax Rice bran wax Candelilla wax Carnauba Brazilian wax Carnauba wild wax Fruit wax Berry wax	Rice bran oil	Polarized light microscopy	Needle-like crystals	Beeswax in rice bran oil exhibited two types of crystals: needle-like crystals and spherulite crystals	[26]
Sunflower wax Berry wax	Rice bran oil	Polarized light microscopy Cryo-scanning electron microscopy	Needle crystals	The berry wax oleogels present higher transparency in their appearance due to the smaller size of the microcrystallites of berry wax oleogel	[94]
Sunflower wax	Canola oil	Polarized light microscopy Cryo-scanning electron microscopy	The PLM indicated the formation of needle-like crystals, while the cryo-SEM proves the formation of the platelets	-	[189]
Wax esters	Medium-chain triglycerides	Bright-field microscopy	Crystals of different sizes with angles of 110° and 70°	The total carbon number and molecular symmetry of the respective wax ester play an important role in the regularity and face length of the formed crystals	[4]
Whey protein	Castor oil, cod liver oil, corn oil Flaxseed oil MCT Peanut oil, sunflower oil	Optical microscopy	The microstructures are less dense, agglomerated, and fine-stranded because of the stabilization of the proteins close to isoelectric pint	The obtained microstructure is characteristic of the unfolding behavior of the globular proteins during the gelation process	[190]
Whey protein	High-oleic soybean oil	Polarized light microscopy	Protein aggregates were observed in the microstructure	Formation of whey protein aggregates is dependent on the concentration of the protein	[191]
White and yellow beeswax Candelilla wax Sunflower wax Monoacylglycerols	Rapeseed oil	Polarized and bright-field light microscopy	“Needle” networks of fatty crystals in structured oils	In the monoacylglycerol oleogels, the formed clusters were the largest	[192]
β-sitosterol and lecithin	Sunflower oil Liquid coconut oil Solid coconut oil	Optical and polarized optical microscopy	Spherulite-like crystals were obtained when used the sunflower oil Sparse spherulite crystals for the liquid coconut oil Radiolitic crystal structure for the case of solid coconut oil	Addition of lecithin promoted formation of needle-like crystals in the case of sunflower oil or decomposition of the radiolitic crystals into small fibrillar radiolitic crystals	[193]
β-sitosterol and stearic acid	Virgin olive oil Corn oil Sunflower oil Flaxseed oil	Optical microscopy	Crystals of different shapes	The shape of the crystals was influenced by the gelator’s concentration, composition, and polarity of the oil	[194]
Agar	Rice bran oil and stearyl alcohol	Bright-field microscopy	Globular-shaped particles dispersed in continuous matrix of agar gel	The globular structures might be attributed to the self-aggregated agar polymeric chains	[41]
Chitosan crosslinked with vanillin	Refined canola oil	Polarized light microscopy	Packed network with crystals of vanillin, depending on the used concentration	The presence of Tween60 (polysorbate) leads to unstable oleogels	[55]

**Table 3:** Rheological results for different used oleogelators and oils.

Oleogelators	Oil	Results	Comments	Reference
Beeswax (BW)	MCT oil	When increasing the concentration to 10%, all the oleogels form a strong gel	BW is the strongest and elastic at 15%	[208]
Monoglyceride (MG)	MCT oil		Weak gel	
Rapeseed wax (RAW)	MCT oil		At any concentration, RAW was not able to form stable structure with MCT oil	
Rice bran wax (RW)	MCT oil		At 10%, it is a weak gel, but at 15%, it is elastic	
Sunflower wax (SW)	MCT oil		Brittle structure	
γ-oryzanol and β-sitosterol mixture	MCT oil		Increasing to 15% did not show modification	
Candelilla wax (CDW)	Grapeseed oil	Form a harder gel	Greater viscoelasticity	[56]
Glyceryl monostearate (GMS)				
Candelilla wax	Canola oil	All the oleogel combinations form an elastic gel	Depending on the temperatures, the gels are more or less elastic	[209]
Carnauba wax	Canola oil			
Beeswax	Canola oil			
Sunflower wax (SW)	Rice bran oil (RBO)	Highest cross-over point	Highest critical stress Good tolerance of deformation	[26]
Beeswax (BW)		Strong gel property	Highest critical stress Good tolerance of deformation	
Rice bran wax (RBW)		Weak and instability network formation	Weak gel strength Unstable at low amplitudes	
Candelilla wax (CLW)		Strong gel property	Larger structural sensitivity	
Carnauba Brazilian wax (CRBW)		Strong gel property	Unstable at low amplitudes	
Carnauba wild wax (CRWW)		Strong gel property	Good tolerance of deformation	
Fruit wax (FW)		Weak and instability network formation	Weaker gel strength Unstable at low amplitudes	
Berry wax (BEW)		Strong gel property	Larger structural sensitivity	
Ethylcellulose (EC)	Sunflower oil	Did not gel	EC alone cannot form gel	[184]
Stearic acid (ST)		Weaker gel	Weak network	
Octadecanol (OD)		Weak gel	For multi-component food	
Monoglyceride (MG)		Weak gel		
Beeswax (BW)		The highest stress resistance	Best structure gel	
Rice bran wax (RW)		Less gel strength	-	
Sunflower wax (SW)		Hardness network	Undergoing changes at storage time	
Mixture of γ-oryzanol and β-sitosterol (γ-β)		The highest stress resistance	Hard gel structure	
Berry wax (BEW)	Rice bran oil (RBO)	Strong molecular network	Weaker gel strength Good tolerance of deformation	[94]
Sunflower wax (SW)		Strong molecular network More brittle than BEW	Better stability under applied stress Good tolerance of deformation Higher stability in the structure than BEW	
Whey protein	Castor oil, cod liver oil, corn oil, flaxseed oil, MCT oil, peanut oil, sunflower oil	The oleogels showed a gel-like behavior, except for the one formulated with castor oil that lacked structuration	The type of oil did not have a major impact on the oleogel networking	[190]
Zein protein	Oleic acid	The results indicate formation of gels	For the gelation is necessary a high amount of ethanol–water	[66]
Colloidal solution of gelatin, tannic acid, and flaxseed gum	Soybean oil	Thermally stable oleogels that have the thixotropic recovery ability at high temperature	The obtained oleogel is appropriate for use in baked products	[210]
Xantham gum	High-oleic sunflower oil	The oleogels have high mechanical strengths	Addition of soy lecithin led to the lowering of the firmness of the oleogel	[211]

**Table 4:** Oxidative stability of some oleogels.

**Organogelator**	**Oil**	**Method**	**Results**	**Comments**	**Reference**
Candelilla waxCarnauba waxBeeswax	Canola oil	Measurement of the peroxide value for 18 days, accelerated conditions (60 °C)	The measured peroxide values for oleogels were higher than those for the fresh oil because of the higher temperature used for the oleogel preparation	The peroxide values increased over time for fresh oils and oleogels, but a faster rate of increasing was observed for fresh oils.The oxidative stability of the oleogels is influenced by the type of used wax: the peroxide value for candelilla wax < carnauba wax < beeswax.This behavior is correlated with the hardness of the oleogel	[209]
Sodium caseinateXanthan gum	Canola oil	Measurement of the peroxide and 2-thiobarbituric acid values	The measured values were similar for oleogel and the fresh canola oil	The xanthan gum, as a biopolymer, contributed to the protection of the oil, which could not be exposed to the prooxidants and oxidation	[213]
Sorbitan monostearate, Soy lecithin, Glyceryl monostearate	Palm olein	Differential scanning calorimetry	The thermal stability of the oleogel is directly proportional to the concentration of the organogelators	The lowest thermal stability was registered for the sorbitan monostearate	[214]
EthylcelluloseBeeswax	Mixture of olive, linseed, and fish oils	Measurement of the thiobarbituric acid values	For the oleogel formulated with ethylcellulose was registered the highest lipid oxidation because of the higher processing temperature and time during the obtaining of the oleogel	The measured oxidation values for both oleogels were below the values that contribute to the detection of unpleasant flavors	[89]
Monoglycerides	High-oleic sunflower oil	Measurement of the peroxide value	The measurement of the peroxide values for 8 weeks indicated low values for all samples	The use of monoglycerides as organogelators improved the oxidative stability of the high-oleic sunflower oil due to the obstruction of oxygen to the active sites by the monoglycerides network	[177]
Beeswax	Olive oil	Measurement of the peroxide value	The oxidative stability was measured for 90 days for samples kept at room and refrigerator temperature, and the results showed that all formulations were stable	The formulated oleogels might be compatible with commercial applications	[179]
Carnauba wax	Canola oil	Measurement of headspace oxygen content, conjugated dienoic acid, p-anisidine content, and headspace volatile compounds	The carnauba wax oleogels showed better oxidative stability than beeswax oleogels, at both studied temperatures 60 °C and 180 °C	The study indicates that carnauba wax contributes to the enhancing of the oleogel oxidative stability and that beeswax is an efficient oleogelator	[91]
Beeswax	Grapeseed oil				

**Table 5:** Textural results for different used oleogelators and oils.

Oleogelators	Oil	Type ofAnalysis	Results	Comments	Reference
Rice bran wax (RBW)	Soybean oil (SBO)	Texture analyzer	Hard texture Good resistance to deformation	The main role in the texture of oleogels is represented by cooling rate	[186]
Candelilla wax (CLW)	Canola oil	Texture analyzer	Highest hardness Greater gumminess	The lowest adhesiveness and cohesiveness	[209]
Carnauba wax (CRW)			Second highest hardness	More gumminess than beeswax	
Beeswax (BW)			The most adhesive and cohesive	Third highest hardness	
Cetyl-derived wax esters	Extra virgin olive oil (VOO)	Texture analyzer	With 60%, the firmness was decreased	Stable texture	[183]
Beeswax (BW)	Sunflower oil (SFO)	Texture analyzer	Structured by fractions, showed the lowest firmness	In combinations of fractions has the highest firmness	[71]
Monoglycerides (MG)	High-oleic sunflower oil (HOSO)	Texture analyzer	Great stability texture just in the first 3 weeks of storage	Hardness decreases over time	[177]
β-Sitosterol (Sit) and Stearic acid (SA)	Olive oil (OLO)	Texture analyzer	Third highest firmness	The gelator mixture and the concentrations of all the oleogels affect the firmness and gel network formation	[194]
	Corn oil (CRO)		Second highest firmness		
	Sunflower oil (SFO)		The lower firmness		
	Flaxseed oil (FSO)		The highest firmness		
Beeswax (BW)	Camellia oil (CO)	Texture analyzer	At 3%, the firmness was lower, and at 2%, it was a flowing liquid	For all oleogels, the storage temperature and oil type can affect the textural parameters	[178]
	Soybean oil (SO)		At 2%, no gel formed		
	Sunflower oil (SFO)		At 2%, no gel formed		
	Flaxseed oil (FO)		The firmness was much greater than that of the others		
Beeswax (BW)	Sunflower oil (SO)	Texture analyzer	Spreadable texture	Satisfactory plastic	[180]
	Olive oil (OO)		Spreadable texture	Satisfactory plastic	
	Linseed oil (LO)		No spreadable texture	Very soft	
	Fish oil (FO)		Spreadable texture	Satisfactory plastic	
Glyceryl monostearate (GM)	Chia seed oil (ChSO)	Texture analyzer	The highest hardness The highest springiness	The highest gumminess Strong gel and more resistant	[207]
Sorbitan monostearate (SM)			The highest cohesiveness	Good texture gel	
Colloidal solution of gelatin, tannic acid, and flaxseed gum	Soybean oil	Texture analyzer	Oven-dried oleogels are spreadable, while freeze-dried oleogels have higher hardness	Different textures observed for the freeze-dried and oven-dried oleogels due to different dehydration conditions	[210]
Chitosan crosslinked with vanillin	Refined canola oil	Texture analyzer	Hardness and adhesiveness were stable for 30 days	Presence of Tween60 affects the texture after 15 days	[55]

## Data Availability

Not applicable.
